# Second-Generation Genetic Linkage Map of Catfish and Its Integration with the BAC-Based Physical Map

**DOI:** 10.1534/g3.112.003962

**Published:** 2012-10-01

**Authors:** Parichart Ninwichian, Eric Peatman, Hong Liu, Huseyin Kucuktas, Benjaporn Somridhivej, Shikai Liu, Ping Li, Yanliang Jiang, Zhenxia Sha, Ludmilla Kaltenboeck, Jason W. Abernathy, Wenqi Wang, Fei Chen, Yoona Lee, Lilian Wong, Shaolin Wang, Jianguo Lu, Zhanjiang Liu

**Affiliations:** The Fish Molecular Genetics and Biotechnology Laboratory, Department of Fisheries and Allied Aquacultures, Program of Cell and Molecular Biosciences, Aquatic Genomics Unit, Auburn University, Auburn, Alabama 36849

**Keywords:** catfish, linkage map, physical map, genome, map integration

## Abstract

Construction of high-density genetic linkage maps is crucially important for quantitative trait loci (QTL) studies, and they are more useful when integrated with physical maps. Such integrated maps are valuable genome resources for fine mapping of QTL, comparative genomics, and accurate and efficient whole-genome assembly. Previously, we established both linkage maps and a physical map for channel catfish, *Ictalurus punctatus*, the dominant aquaculture species in the United States. Here we added 2030 BAC end sequence (BES)-derived microsatellites from 1481 physical map contigs, as well as markers from singleton BES, ESTs, anonymous microsatellites, and SNPs, to construct a second-generation linkage map. Average marker density across the 29 linkage groups reached 1.4 cM/marker. The increased marker density highlighted variations in recombination rates within and among catfish chromosomes. This work effectively anchored 44.8% of the catfish BAC physical map contigs, covering ∼52.8% of the genome. The genome size was estimated to be 2546 cM on the linkage map, and the calculated physical distance per centimorgan was 393 Kb. This integrated map should enable comparative studies with teleost model species as well as provide a framework for ordering and assembling whole-genome scaffolds.

Most agriculturally important performance and production traits are controlled by quantitative trait loci (QTL). Understanding and utilizing QTL for genetic improvements requires genetic linkage maps. As such, genetic maps have been constructed in essentially all economically important species. In the last decade, genetic maps have been constructed in various aquaculture species [for review, see Danzmann and Gharbi (2007)]. In most aquaculture species, however, genetic linkage maps have low density of markers so that their utility for the studies of QTL is limited. Recently, high-density genetic maps have been constructed in a number of fish species, such as Atlantic salmon (Lien *et al.* 2011), rainbow trout (Rexroad *et al.* 2008; Miller *et al.* 2012), and Asian seabass (Wang *et al.* 2011). In catfish, genetic linkage maps have been constructed using both intraspecific and interspecific resource families targeted at selective breeding and introgression programs (Kucuktas *et al.* 2009; Liu *et al.* 2003; Waldbieser *et al.* 2001). However, these maps harbor only a couple of hundred markers. One of the aims of this study is to construct a second-generation high-density genetic map.

Map integration aims to place a shared set of markers on both linkage and physical maps such that one can relate genetic map positions and physical sequence. Integrated maps are a valuable resource for fine mapping of QTL, positional cloning of important genes, comparative genome analysis with similar species, and as a framework map for whole-genome assembly (Palti *et al.* 2012; Palti *et al.* 2011). Several methods have been used previously for the integration of the physical and genetic maps, including (1) *in silico* comparison of marker sequences to a whole-genome sequence; (2) BAC pooling and PCR screening; (3) hybridization using overgo probes; and (4) mapping of molecular markers from BAC end sequences (Cordoba *et al.* 2010; Yim *et al.* 2007; Romanov *et al.* 2003; Yuan *et al.* 2000). In the last method, the most widely exploited markers have been microsatellites (Cordoba *et al.* 2010). Integration of linkage maps with BAC-based physical maps has been carried out in some fish species, including medaka (Matsuda *et al.* 2002), barramundi (Wang *et al.* 2008), Atlantic salmon (Lorenz *et al.* 2010), and rainbow trout (Palti *et al.* 2012; Palti *et al.* 2011), although the extent of integration varies among species.

Channel catfish (*Ictalurus punctatus*) is the leading aquaculture species in the United States and an important model of neurobiology, ecotoxicology, and immunology (Jemal *et al.* 2010; Booth and Bilodeau-Bourgeois 2009; Hansen *et al.* 2003). Efforts over the last decade have focused on developing genomic resources [reviewed by Liu (2011)]. Concurrent with the generation of microsatellite-based linkage maps for catfish (Kucuktas *et al.* 2009; Waldbieser *et al.* 2001) and catfish physical maps (Quiniou *et al.* 2007; Xu *et al.* 2007), BAC end sequencing was carried out on the majority of the physical map clones (Liu *et al.*, 2009; Wang *et al.* 2007; Xu *et al.* 2006). The BES collection established a resource for mining polymorphic microsatellites that could serve as a connecting point between catfish genetic and physical maps (Somridhivej *et al.* 2008). In catfish, 17.5% of BAC end sequences (63,387) were found to contain microsatellites (Xu *et al.* 2006). Here, we have utilized this BES resource for genetic mapping, which successfully integrated 1481 physical map contigs with a high-density catfish linkage map, generating a strong foundation for comparative and functional genomics in catfish and a framework for forthcoming reference genome sequence assemblies.

## Materials and Methods

### Resource family

The resource family for BES markers used in this study was previously described (Liu *et al.* 2003). In brief, a F_1_ interspecific hybrid catfish was generated from the most informative mating of a channel catfish female and a blue catfish (*Ictalurus furcatus*) male. In 1997, backcross families were made using F_1_ fish mating with channel catfish (backcross). A specific family, F_1_-2 × Channel catfish-6, was used for this project.

### Genomic DNA isolation

DNA was extracted from 64 samples plus their 2 parents from resource family F_1_-2 × Channel catfish-6. Blood samples (0.5 to 1 ml) were collected in a 1-ml syringe and immediately expelled into a 50-ml tube containing 20-ml of DNA extraction buffer (100 mM NaCl, 10 mM Tris, pH 8, 25 mM EDTA, 0.5% SDS, and freshly added proteinase K 0.1 mg/ml), and DNA was isolated by using the Puregene DNA Isolation Kit (Gentra Systems, Minneapolis, MN).

### Identification of microsatellites, primers, and PCR amplification

Catfish BAC end sequences were generated by Xu *et al.* (2006). BES stored in a local database at the Fish Molecular Genetics and Biotechnology Laboratory and available publicly in the NCBI GSS database and in the cBARBEL database (Lu *et al.* 2011) were used for microsatellite mining. The channel catfish BAC-based physical map, web FPC viewer version 2.1: AU 02-20 (http://titan.biotec.uiuc.edu/WebAGCoL/AU02-20/WebFPC/), was used to obtain clones containing microsatellites (Somridhivej *et al.* 2008).

BES-containing microsatellites with at least 50 bp of flanking sequences on both sides were used for primer design by *Msatfinder*. Primers were designed to amplify product sizes between 100 and 250 bp. However, in some instances, longer PCR products were accepted. A 19-bp tail sequence (GAGTTTTCCCAGTCACGAC) was added to the 5′ end of the upper primer (Oetting *et al.* 1995). A primer whose sequence is complementary to the tail sequence was used as the label [labeled with infrared dye (IRD)-IRD700 or IRD800 from LI-COR Biosciences, Lincoln, NE]. All primers were ordered from Invitrogen (Carlsbad, CA).

PCR reactions were performed on a Mastercycler (Eppendorf, Hauppauge, NY) or on a DNA Engine Thermocycler PTC 200 (Bio-Rad, Hercules, CA) using the following amplification profiles: a 5-µl PCR reaction mixture containing 1 µl of 50 ng/µl genomic DNA (Gentra Puregene kit), 0.5 µl of 10X PCR buffer, 0.2 µl of 50 mM MgCl_2_, 0.4 µl of 2.5 mM dNTP, 0.2 µl of 10 pmol/µl upper primer (with tailed primer 5′GAGTTTTCCCAGTCACGAC3′ added at 5′ end), 0.3 µl of 10 pmol/µl lower primer, 0.1 µl of 1 pmol/µl primer label IRD700 or IRD800, and 0.05 µl of 5 U/µl of Platinum Taq polymerase. PCR amplifications were conducted using 384-well plates. Two-step PCR profiles were used for amplification. An initial denaturation step at 94° for 3.5 min was followed by a first denaturation at 94° for 30 s, first annealing step at 57° for 30 s, and a first extension at 72° for 30 s. This first step was repeated for 20 cycles followed by 15 cycles of a second step with the following parameters: denaturation at 94° for 30 s, annealing at 53° for 30 s, and extension at 72° for 30 s followed by a final extension step at 72° for 15 min. The samples were held at 4° for 15 min. In this two-step PCR profile, the annealing temperature window was accommodated to determine the best T_m_ for the primer pairs. PCR products were analyzed on a 7% polyacrylamide gel using a LICOR 4300 DNA Analyzer (LICOR Biosciences, Lincoln, NE).

### Genotyping

After gel electrophoresis, the polymorphic microsatellite bands were scored and genotyped based on the allele segregation within the resource family. A Chi-square goodness-of-fit test was used to assess the Mendelian segregation patterns. Genotype configurations of markers can be categorized into three expected segregation patterns when null-allele segregation was allowed: 1:1:1:1-ratio type (♀ × ♂: AB × CD or AB × AC), 1:1 ♀ type (AB × AA or CC), and 1:1 ♂ type (AA or CC × AB).

### Microsatellite markers, type I microsatellites, microsatellites derived from next-generation sequencing, and SNP markers

Locus identifications and genotyping of anonymous microsatellites, type I EST, and SNP markers were previously described in Waldbieser *et al.* (2001) and Kucuktas *et al.* (2009), respectively. Identification of microsatellites by next-generation sequencing (NGS) of the catfish genome was used for generating several NGS markers.

### Linkage analysis

All linkage analysis was made using JoinMap version 4.0 software (Van Ooijen 2006). Linkage between markers was examined by estimating LOD scores for recombination rate (*Θ*), and map distances were calculated using the Kosambi mapping function. Significance between linkage groups was determined using a LOD threshold of 14.0, and a threshold *Θ* of 0.6 was set to detect suspect linkage possibly resulting from allele-coding errors. The high LOD score was used to provide high levels of confidence for the correctness of the marker locations.

Markers were linearly aligned in each linkage group, converting recombination rates into Kosambi’s map distance (centimorgans). The position of markers was explored on the basis of the sequential buildup of the map (Stam 1993). First, the most informative pair of markers was selected, followed by sequential addition of other markers. The ‘‘ripple’’ was performed each time after adding one marker. The best fitting position of an added marker was searched on the basis of the goodness-of-fit test (chi-square) for the resulting map. When a marker generated a negative map distance in the map or a large ‘‘jump’’ value in goodness-of-fit, which is the normalized difference in chi-square value before and after adding the marker, the marker was removed, and map calculation was continued to construct a first-round map. After the first-round marker ordering, the previously removed markers were added to the first-round map and again subjected to the goodness-of-fit testing. In this manner, the marker ordering was continued up to the third round until an optimum order of markers was found.

## Results

### Microsatellite markers from physical map–associated BAC end sequences

We screened 30,582 BAC end sequences on the catfish physical map for microsatellites useful for linkage mapping and map integration. The catfish physical map derived from fluorescent fingerprinting of the CHORI-212 BAC library contains 3307 contigs (Xu *et al.* 2007). Ideally, at least one microsatellite from each of the 3307 contigs, possibly more than one microsatellite for large contigs (for orientation), is needed to fully anchor the physical map contigs to the linkage map. Our screening identified high quality microsatellites in BES associated with 2128 of the physical contigs. The remaining physical map contigs could not be targeted by this approach, either due to lack of BES-associated microsatellites or microsatellites lacking sufficient flanking regions for primer design. A total of 3846 microsatellites from the targeted physical map contigs were selected for PCR optimization and genotyping. Of these, 2116 BES microsatellites from 1534 unique physical map contigs amplified a single product of expected size and could be resolved and unambiguously scored (Table 1). These BES microsatellite markers were carried forward for use in construction of the genetic linkage map.

**Table 1:** Development of physical map contig-specific microsatellites from BAC end sequences

Item	Number
Contigs	3307
Contigs with microsatellites	2128 (64.3% of total)
Contigs with polymorphic microsatellites	1534 (72.1% of contigs with microsatellites)
Polymorphic microsatellites in the 1534 contigs	2116
Contigs with two or more polymorphic microsatellites	385

### Linkage mapping

To construct a high-density second-generation genetic linkage map in catfish, a total of 5543 markers were tested in our interspecific hybrid resource family, F_1_-2 × Channel-6 (Liu *et al.* 2003), resulting in 2740 markers polymorphic within this resource family (see Table S3 for primer information of the mapped microsatellites, and Table S4 for primer information of the unmapped microsatellites). The 2740 polymorphic makers included 2116 BES microsatellites derived from the physical map contigs, 77 BES markers from BAC singletons, 100 SNP markers, 283 EST markers (Kucuktas *et al.* 2009), 147 anonymous microsatellite markers (Waldbieser *et al.* 2001), and 17 microsatellites derived from next-generation sequencing (Table 2). The EST and SNP markers were utilized in the framework linkage map genotyped on the same resource family (Kucuktas *et al.* 2009), whereas the 147 anonymous microsatellites were utilized for construction of a framework linkage map using an intraspecific resource family (Waldbieser *et al.* 2001). To start the linkage analysis of the sex-averaged map, the LOD score was set at 14.0 in order to generate the linkage groups based on the segregation data. The high LOD score was used to correctly assign the markers to their linkage groups. Among 2740 polymorphic markers, 2557 markers, including 2099 BES-based microsatellites, 235 EST-derived microsatellites, 127 anonymous microsatellites, 17 next-generation sequencing-based microsatellites and 79 SNPs were mapped and assigned to 29 linkage groups. A total of 93.3% of genotyped markers could be successfully mapped (Table 2 and Figure S1). The remaining 183 markers were not mapped because of significant segregation distortion, unlinked markers, or other mapping abnormalities.

**Table 2:** Summary of markers used for construction of the genetic linkage map

Marker Type	Attempted	Polymorphic	Mapped
Microsatellites from BES	3846	2193	2099
SNPs	384	100	79
Microsatellites from EST	1025	283	235
Microsatellites from anonymous genomic locations (IpCG)	268	147	127
Microsatellites from NGS	20	17	17
Total	5543	2740	2557

The sum of intervals from all loci of the sex-averaged map after excluding stacked markers was 2467.9 cM (Kosambi). To correct the chromosome ends (telomeric regions), the estimated total map size was calculated by adding 2x, where x is the average space between adjacent markers, to all map sizes of each linkage group (Fishman *et al.* 2001). Therefore, the estimated corrected map size of the sex-averaged map was approximately 2550 cM. The average number of mapped markers per LG was 63 markers, and ranged from 31 to 87 markers. Intermarker distance varied from 0 to 43.7 cM (Table 3).

**Table 3:** Characteristics of the catfish genetic map with 2557 markers at 1836 unique map positions among the 29 linkage groups

Linkage Group	Unique Markers	Map Size (cM)	Total Map Size (cM)	Average Marker Density (cM)	Range of Intermarker Spacing (cM)
1	72	76.6	79.3	1.1	0–12.9
2	72	79.9	82.6	1.1	0–6.6
3	80	99.9	102.6	1.2	0–14.9
4	67	88.3	91.0	1.3	0–17.1
5	75	70.0	72.7	0.9	0–6.6
6	50	71.6	74.3	1.4	0–17.6
7	57	78.3	81.0	1.4	0–15.9
8	80	127.8	130.5	1.6	0–43.7
9	59	70.3	73.0	1.2	0–13.0
10	62	98.0	100.7	1.6	0–13.2
11	62	83.9	86.6	1.4	0–22.6
12	80	94.5	97.2	1.2	0–10.0
13	78	114.2	116.9	1.5	0–11.7
14	70	87.0	89.7	1.2	0–13.0
15	80	93.7	96.4	1.2	0–12.6
16	76	111.6	114.3	1.5	0–11.4
17	31	14.1	16.8	0.5	0–2.0
18	52	73.6	76.3	1.4	0–21.8
19	51	90.9	93.6	1.8	0–10.9
20	87	91.0	93.7	1.0	0–13.5
21	65	73.2	75.9	1.1	0–8.9
22	49	89.2	91.9	1.8	0–15.1
23	55	87.7	90.4	1.6	0–12.6
24	43	69.2	71.9	1.6	0–15.7
25	64	84.5	87.2	1.3	0–7.4
26	59	57.5	60.2	1.0	0–5.2
27	52	100.8	103.5	1.9	0–6.0
28	71	93.4	96.1	1.3	0–8.8
29	37	97.2	99.9	2.6	0–18.3
Total	1836	2467.9	2546.2	1.4	—

### Marker distribution and map characteristics

**Figure 1:**
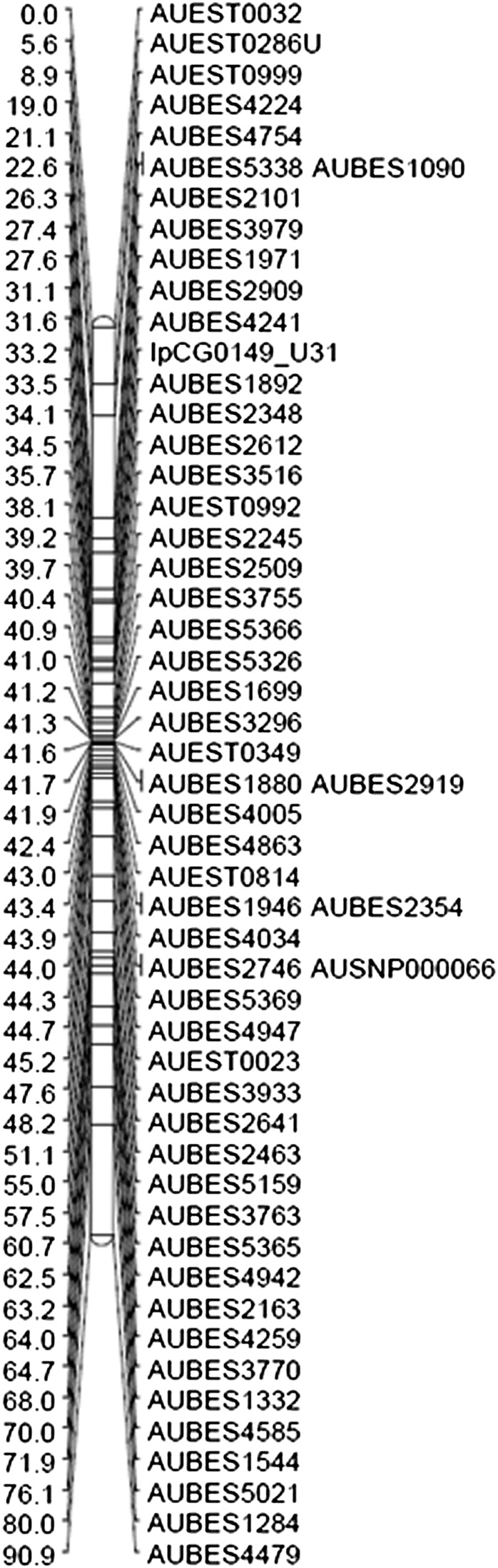
An example linkage group (LG19) from the genetic linkage map of channel catfish. Genetic map distance is given in centimorgans (Kosambi’s mapping function) to the left of the markers positions. The vertical straight lines indicate markers placed at the same position.

In the resulting genetic linkage map, markers were distributed across all 29 linkage groups with a minimum of 46 markers/LG (LG29; see Figure 1 for a representative LG). Similarly, the linked physical map contigs were distributed across linkage groups, ranging from 33 to 77 contigs/LG (Table 4). However, distribution of markers was uneven, likely reflecting differences in recombination frequency along the length of the catfish chromosomes (Figure S1). Clustered marker regions were observed in every linkage group of the sex-averaged linkage map, especially in positions close to the centromeres and, less frequently, at the telomeres.

**Table 4:** Characteristics of marker distribution among 29 linkage groups

Linkage Group	Mapped Markers	Contigs	Stacked Markers	Stacked Marker Positions	Unique Marker Positions
1	113	77	56	15	72
2	84	49	19	7	72
3	107	74	41	14	80
4	104	70	48	11	67
5	98	59	33	10	75
6	126	74	89	13	50
7	89	47	44	12	57
8	119	75	55	16	80
9	91	50	43	11	59
10	68	36	11	5	62
11	89	60	36	9	62
12	101	58	29	8	80
13	100	57	32	10	78
14	81	43	18	7	70
15	101	52	33	12	80
16	98	55	32	10	76
17	93	52	67	5	31
18	71	45	25	6	52
19	55	41	8	4	51
20	121	73	44	10	87
21	74	43	15	6	65
22	80	50	39	8	49
23	78	52	32	9	55
24	60	38	22	5	43
25	87	57	31	8	64
26	76	42	27	10	59
27	65	35	21	8	52
28	82	47	21	10	71
29	46	33	14	5	37
Total	2,557		985	264	1,836

To characterize marker clustering, we tabulated the number of markers sharing an identical genetic map position with other markers, as well as the number of map positions where markers were “stacked” or redundant (Table 4). Linkage group 17 had the highest percentage (72%) of markers placed at the same location, whereas linkage group 19 had only 14.5% of its markers at stacked map positions (Table 4 and Figure 1). To obtain a more accurate evaluation of marker distribution and map density, we calculated the number of unique markers per linkage group by subtracting the markers at stacked positions (identical centimorgan location) and adding back the number of stacked marker positions (to account for one unique marker per stacked position). A total of 1836 unique marker positions remained. We then utilized unique marker counts for each linkage group along with estimated linkage group sizes (with corrections for telomere ends) to calculate an average LG density (cM/marker). Densities ranged from 0.5 cM/marker in LG17 to 2.6 cM/marker in LG29, with an average of 1.4 cM/marker. Of note, however, is the skewed corrected map size of LG17 at only 16.8 cM, likely reflecting the severe shortage of recombination events observed in this linkage group. In contrast, the telomere-corrected average size of the 29 catfish linkage groups was 87.8 cM (Table 3).

### Evaluation of map integration

Of the 1534 physical map contigs with at least one successfully genotyped BES marker (Table 1), 1481 physical map contigs, approximately 69.6% of physical map contigs with microsatellites, were successfully integrated with the linkage map (Table 5). Due to multiple BES markers being successfully mapped from some physical map contigs, a total of 2,030 contig-based BES were placed in the linkage map. Detailed information regarding mapped contig identities, estimated physical contig sizes, corresponding linkage groups, and genetic map positions is listed in Table S1. In terms of the physical coverage of the integrated genetic map, the integrated physical map contigs include a total of 17,972 individual BAC clones.

**Table 5:** Assessment of integration of the catfish physical and linkage maps

Item	Number
Contigs with polymorphic microsatellites	1534 (46.4% total contigs)
Contigs mapped to linkage map	1481 (96.5%)
BES microsatellite markers mapped from the 1481 contigs	2030
Contigs mapped with only one microsatellite marker	1096
Contigs with two or more microsatellites mapped	385
Contigs with two or more microsatellites mapped at the same position	191
Contigs with two or more microsatellites mapped at adjacent map positions	140
Contigs with two or more microsatellites mapped in different linkage groups	54
Average length of integrated physical map contigs (kb)	344
BAC clones contained in the 1481 contigs	17,972
BAC clones on the catfish physical map of 3307 contigs	30,582
% of BAC clones on the physical map integrated with linkage map	58.8%
Total length of integrated physical map contigs (kb)	510,074

**Figure 2:**
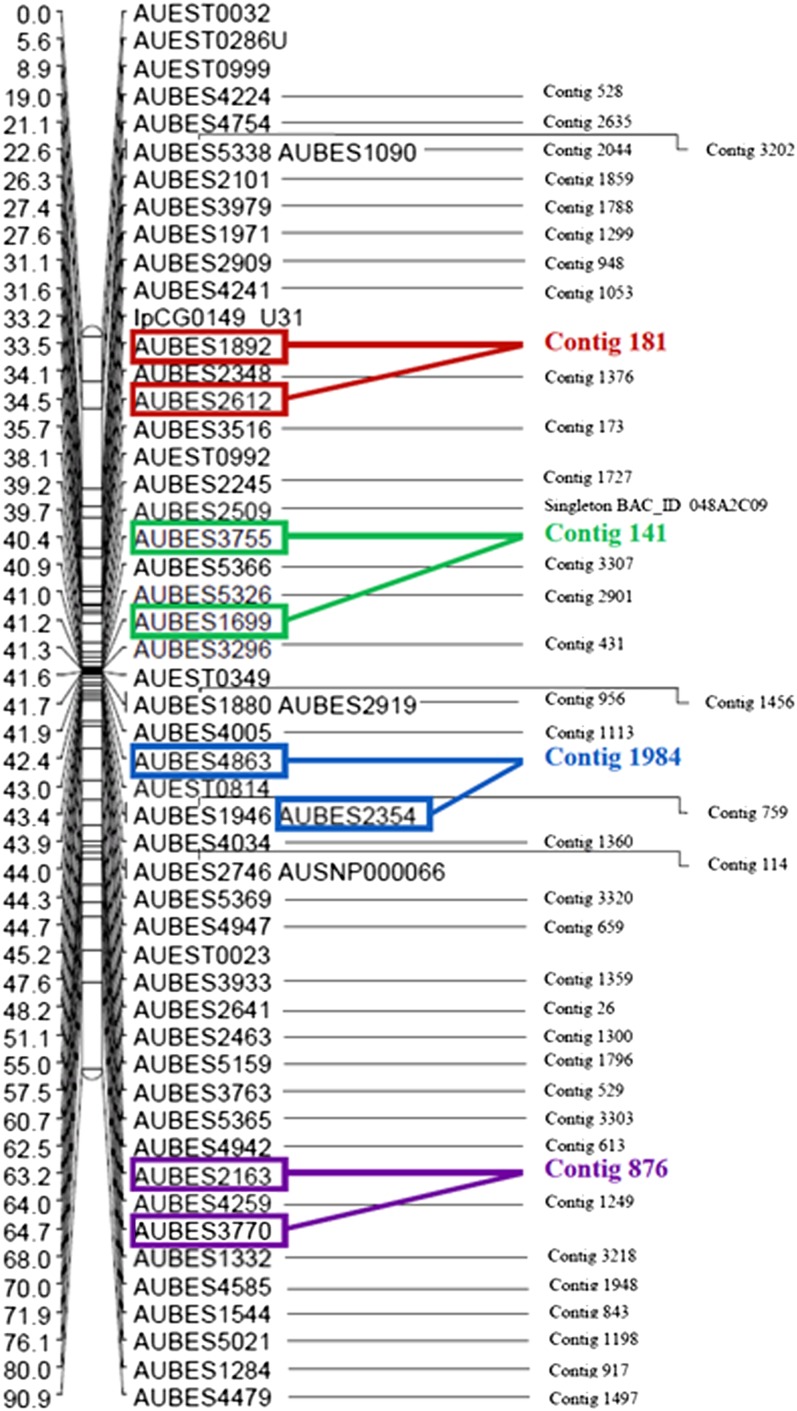
An example linkage group with physical map contig identities integrated (LG19) via their BES markers. Square boxes of the same color indicate markers mapped from the same contig.

In most cases, markers derived from the same physical contigs were mapped in the same or adjacent linkage position, reflecting the proximity of the markers physically. However, 54 physical map contigs, each with at least two BES markers, were mapped to multiple linkage groups based on conflicting assignments of individual BES markers, suggesting that these physical map contigs may be erroneously constructed or that they fall within duplicated regions of the genome (Table 5). An example of a linkage group with physical map contig information integrated based on marker position is illustrated in Figure 2.

Given an estimated haploid genome size of channel catfish and blue catfish of approximately 1×10^9^ bp (Tiersch and Goudie 1993; Tiersch *et al.* 1990), and a total corrected map size of 2546 cM, we calculated the relationship between physical and genetic map distances to be approximately 393 kb/cM. Additionally, the combined estimated physical length of the 1481 anchored physical map contigs was equal to 510,074 Kb (Table 5), leading us to calculate that the integrated map covered ∼45% of the physical map contigs and ∼52.8% of the estimated catfish genome. Anchored contigs varied in size from 88 Kb to 2206 Kb, with an average size of 344 Kb (Table S1).

A total of 322 contigs were mapped with multiple markers and observed recombination between at least one pair of markers, allowing contig-based estimations of ratios of physical to genetic distance (Table S2). Physical distances were estimated based on position of the BES markers within each contig. Multiple marker contigs were distributed across all 29 LGs. The average physical distance per centimorgan calculated from these observed markers with recombination was slightly larger than the whole-genome average.

## Discussion

The aim of this study was to construct a high-density genetic linkage map using BAC end sequence-derived microsatellites to integrate a BAC-based physical map of channel catfish with genetic linkage maps. In the process, we sought to also increase the marker density and utility of the catfish genetic linkage map for genomic studies. We mapped 2030 BES markers from 1481 physical map contigs to integrate a substantial portion of the physical map and to boost average marker density to 1.4 cM/marker. As the major objective is to increase the marker density on the map, here we reported a sex-averaged map rather than sex-specific map. However, we are aware that recombination rate is higher in the female map than in the male map, approximately 1.6:1.0 as we previously reported (Kucuktas *et al.* 2009), which is less dramatic than the situation in salmonids (*e.g.*, Moen *et al.* 2008).

Map integration is a critical component of efforts aimed at marker-assisted selection in aquaculture species. Fine QTL mapping is often thwarted by an inability to identify genomic regions containing causative genes and/or additional markers in linkage disequilibrium with the locus of interest. With an integrated map, markers of interest can be related to BAC contigs, which can then be quickly sequenced using next-generation sequencing approaches to obtain the genic content and structural organization. Additionally, whole-genome assembly can be significantly enhanced using an integrated map to orient and order sequence scaffolds along the length of linkage groups based on their matches with BES, allowing generation of chromosome-scale scaffolds. Significant gaps revealed during this process can be filled by targeting BAC clones within the gaps for further sequencing. Furthermore, prior to whole-genome assembly, information captured through ordering of physical map BES can be used for comparative genomics studies (Palti *et al.* 2012; Yu *et al.* 2009; Liu *et al.* 2009).

The utility of genetic and integrated maps is directly correlated with the even distribution of markers across linkage groups. Clustered markers in areas of minimal recombination, while allowing general linkage group assignment, often cannot be used for definitive fine mapping and positional cloning. Physical map contigs placed within these regions similarly cannot be ordered and oriented in relation to other nearby contigs. In fish, clustering of DNA markers on genetic linkage maps has been observed in medaka (Naruse *et al.* 2000), rainbow trout (Robison *et al.* 2001), tilapia (Kocher *et al.* 1998), channel catfish (Liu *et al.* 2003), and Atlantic salmon (Lorenz *et al.* 2010). Although potential explanations for high levels of marker clustering are not completely understood (Watanabe *et al.* 2005), most are based on dead zones of recombination. Numerous studies have reported large disparities between genetic map and physical map distances (*e.g.* Espeso *et al.* 2005; Gustafson *et al.* 1990; Gustafson and Dille 1992; Young and Tanksley 1989). This phenomenon is often attributed to differences in genic content across chromosomes, with recombination decreasing in chromosomal regions containing heterochromatin, large areas of repetitive DNA, and in regions around centromeres and/or telomeres. The catfish genome is known to have long repetitive regions, including Xba elements and abundant transposable elements that likely interfere with recombination (Jiang *et al.* 2011; Liu *et al.* 1998; Nandi *et al.* 2007; Xu *et al.* 2006). We observed significant clusters of markers on the majority of the catfish linkage groups. However, some linkage groups were clearly more impacted by this phenomenon than others. For example, linkage group 17 had 67 markers (out of 93 total) in 5 stacked-marker positions. It is noteworthy that an interspecific resource family is being used in this study, and chromosomal-level homologies may vary among chromosomes. It is possible that the linkage groups with extremely high-clustered markers may indicate a lower level of chromosome homology between channel catfish and blue catfish.

Several other potential reasons for low recombination in these regions deserve exploration. The number of genotyped progeny may impact rates of observed recombination between genetic markers. We examined whether the use of 64 fish for genotyping provided insufficient power to detect rare recombinants. We genotyped 128 fish (64 original plus 64 additional family members) for 93 BES markers found within highly clustered map regions. Recombination rates rarely increased substantially from those seen using 64 fish (data not shown), indicating that progeny number was not limiting in these cases. However, significant increase in progeny numbers should increase resolution, allowing detection of rare recombination although the involved cost would be high.

A hybrid system was used in this study for mapping because channel catfish and blue catfish each harbor a set of superior production and performance traits. Channel catfish exhibits superior growth, feed conversion efficiency, resistance to the bacterial disease caused by *Flavobacterium columnare*, and tolerance to low oxygen. Blue catfish exhibits superior performance for processing yields, seinability, and resistance to the most serious bacterial disease, enteric septicemia of catfish (ESC) caused by *Edwardsiella ictaluri*. The channel × blue hybrids exhibit heterosis for almost all economically important traits. However, mass production of the hybrid has been difficult due to problems involved in artificial spawning. Mapping studies using the hybrid system should allow understanding of superior traits in both species to facilitate genome-based selection and introgression strategies.

It has also been long noted that recombination is reduced in hybrids due to decreases in homology and reduced pairing of chromosomes during meiosis (Kreike and Stiekema 1997; Young and Tanksley 1989). This leads in turn to linkage drag where large, nonrecombining fragments are retained in backcrosses, an issue long confronted by plant breeders (Rhyne 1962). The use of a hybrid backcross family in the present study, therefore, may have increased the extent and size of cold zones of recombination. In the future, genotyping of clustered markers on an intraspecies mapping panel may increase resolution within at least some of the low recombination regions. The clustered BES markers, while of minimal utility in fine ordering of physical map contigs for QTL analysis within these regions or assisting in whole-genome assembly, do provide gross assignment of physical map contigs to linkage group regions.

The ratio of physical to genetic linkage distances varied substantially across the 29 linkage groups and between markers within the same physical map contig, again illustrating differing rates of recombination across the catfish genome, as seen in other vertebrate genomes (Groenen *et al.* 2009; Quiniou *et al.* 2007; Nievergelt *et al.* 2004; Nachman 2002). Ratios calculated on a linkage group level are impacted by the presence of non-BES markers in the analysis. These markers (ESTs, SNPs, and others) contribute to the genetic map distance, but they are not part of the physical distance estimation based on contig lengths and thereby skew the calculated ratios. To avoid this issue, we focused on physical to genetic distance ratios based on multiple markers mapped from the same contigs (Table S2). Ratios ranged from 4 kb/cM, potentially indicating hyper-recombination, to 4830 kb/cM, indicating suppressed recombination. Several contigs showed no recombination between markers.

A combination of approaches will be utilized for integration of the remaining 1826 contigs not integrated in the present study because of lack of sufficient microsatellite sequences or insufficient polymorphism in existing microsatellites. For some contigs, we are in the process of identifying SNPs within BES regions (Lorenz *et al.* 2010) for mapping. Other contigs will be integrated through connection of BES sequences with SNP-containing genomic regions via initial catfish genome assemblies. These SNPs in turn will be genotyped on large reference families utilizing a high-density Affymetrix SNP chip. As the catfish genome assemblies improve, additional contigs will be integrated through the physical sequence connection of genotyped markers and contig BES. Recent rapid improvement in both sequencing and SNP genotyping technologies presents many new options for achieving full integration of the catfish genome resources (Liu *et al.*, 2011; Scaglione *et al.* 2012; Davey *et al.* 2011; Van Oeveren *et al.* 2011; Baird *et al.* 2008).

Although variation in recombination frequencies complicates the relationship between genetic and physical distance, integrated maps are valuable for checking for errors in individual map assemblies. The majority of contigs with multiple markers were placed into a single linkage group. However, 54 anchored contigs were mapped to multiple linkage groups. This could indicate FPC assembly error during physical map construction, resulting in mis-assembly of genomic regions. Increasing stringency of assembly of these regions may resolve the issue. However, in some cases, these contigs may be the result of assembly of duplicated regions of the catfish genome that share high levels of sequence similarity (Xu *et al.* 2007), at least at the relevant restriction sites used for the construction of the catfish physical map. The “breaking” of these contigs during linkage mapping is of great utility in highlighting these potentially duplicated regions. We examined whether increasing stringency for FPC construction of the physical map could resolve the assembly in problematic contigs. We have increased the cutoff values for the contig assembly to 1×10^−25^ and 1×10^−30^, respectively. The majority of problematic contigs (37 out of 54) were split from the original contigs into different contigs or singletons when increasing the threshold, while 17 of 54 contigs remained in the same physical map contigs. Whether a BAC contig is to be split into different contigs when assembly stringency is increased depends on the level of overlapping segments generated from restriction fingerprinting. Apparently, the 17 contigs that remain in the same contigs with increased assembly stringency indicated that long stretches of duplicated genome segments may have been involved in these contigs. Such information will be important for the assembly of whole-genome sequences. Additionally, in a few instances, contigs whose BES markers span an unexpectedly large genetic map distance (>10 cM), may also indicate mis-assembly, or intrachromosomal duplications. Such large discrepancy between linkage map and physical map distances could also come from genome variations, as the resource family used for genetic linkage map is not related with the fish used for physical mapping. Given the large variation in recombination ratios across the catfish linkage groups, however, these determinations will be more difficult to make at present with the available genome resources.

## Conclusions

The catfish integrated map presented here substantially increases linkage marker density available for QTL studies and connects the majority of the catfish genetic markers to physical sequence BAC contigs through the use of BES microsatellites. The integrated map will allow trait-based studies (*e.g.* Ninwichian *et al.* 2012) to expand beyond linkage analysis to fine mapping and selection of important candidate genes for further research. The integrated map will also be a valuable resource in improving and validating the catfish whole-genome assembly currently under way.

## Supplementary Material

Supporting Information
