# Supplementary material for: Second-Generation Genetic Linkage Map of Catfish and Its Integration with the BAC-Based Physical Map
Source: G3 (Bethesda). 2012 Oct 1;2(10):1233–41. doi: 10.1534/g3.112.003962 (PMC3464116; doi:10.1534/g3.112.003962)
Supplement: Supporting Information [file supp_2_10_1233__index.html]

Supporting Information 

# Second-Generation Genetic Linkage Map of Catfish and Its Integration with the BAC-Based Physical Map

## Supporting Information for Ninwichian *et al.*, 2012

**Files in this Data Supplement:**

- Figure S1 - A sex-averaged linkage map of channel catfish (PDF, 4 MB)
- Table S1 - Detailed information regarding contig identities, estimated physical contig sizes, corresponding linkage groups, and genetic map positions of 2,099 BAC-end sequence mapped microsatellite markers (.xlsx, 209 KB)
- Table S2 - Table S2: Ratios of physical to genetic distance across all 29 LGs. Ratios were estimated based on the difference of the physical distances (Kb) and genetic distances (cM) between multiple BES markers mapped from the same contigs (.xlsx, 27 KB)
- Table S3 - Detailed primer sequence information regarding mapped markers (.xlsx, 119 KB)
- Table S4 - Detailed primer sequence information regarding unmapped markers (.xlsx, 130 KB)
