# Supplementary material for: Second-Generation Genetic Linkage Map of Catfish and Its Integration with the BAC-Based Physical Map
Source: G3 (Bethesda). 2012 Oct 1;2(10):1233–41. doi: 10.1534/g3.112.003962 (PMC3464116; doi:10.1534/g3.112.003962)
Supplement: Supporting Information [file supp_2.10.1233_FigureS1.pdf]

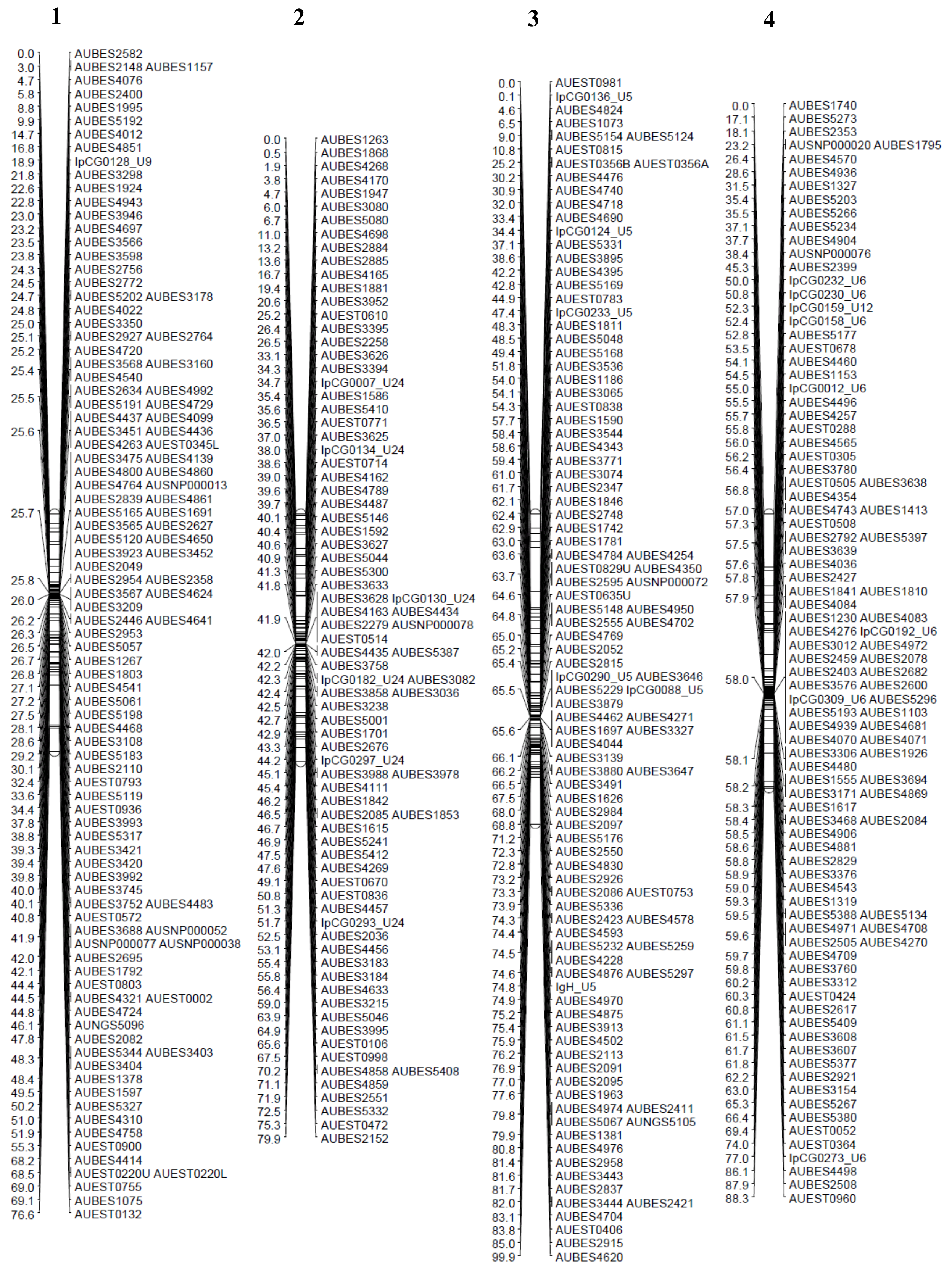
1
0.0
3.0
4.7
5.8
8.8
9.9
14.7
16.8
18.9
21.8
22.6
22.8
23.0
23.2
23.5
23.8
24.3
24.5
24.7
24.8
25.0
25.1
25.2
25.4
25.5
25.6
25.7
25.8
26.0
26.2
26.3
26.5
26.7
26.8
27.1
27.2
27.5
28.1
28.6
29.2
30.1
32.4
33.6
34.4
37.8
38.8
39.3
39.4
39.8
40.0
40.1
40.8
41.9
42.0
42.1
44.4
44.5
44.8
46.1
47.8
48.3
48.4
49.5
50.2
51.0
51.9
55.3
68.2
68.5
69.0
69.1
76.6
AUBES2582
AUBES2148 AUBES1157
AUBES4076
AUBES2400
AUBES1995
AUBES5192
AUBES4012
AUBES4851
IpCG0128_U9
AUBES3298
AUBES1924
AUBES4943
AUBES3946
AUBES4697
AUBES3566
AUBES3598
AUBES2756
AUBES2772
AUBES5202 AUBES3178
AUBES4022
AUBES3350
AUBES2927 AUBES2764
AUBES4720
AUBES3568 AUBES3160
AUBES4540
AUBES2634 AUBES4992
AUBES5191 AUBES4729
AUBES4437 AUBES4099
AUBES3451 AUBES4436
AUBES4263 AUEST0345L
AUBES3475 AUBES4139
AUBES4800 AUBES4860
AUBES4764 AUSNP000013
AUBES2839 AUBES4861
AUBES5165 AUBES1691
AUBES3565 AUBES2627
AUBES5120 AUBES4650
AUBES3923 AUBES3452
AUBES2049
AUBES2954 AUBES2358
AUBES3567 AUBES4624
AUBES3209
AUBES2446 AUBES4641
AUBES2953
AUBES5057
AUBES1267
AUBES1803
AUBES4541
AUBES5061
AUBES5198
AUBES4468
AUBES3108
AUBES5183
AUBES2110
AUEST0793
AUBES5119
AUEST0936
AUBES3993
AUBES5317
AUBES3421
AUBES3420
AUBES3992
AUBES3745
AUBES3752 AUBES4483
AUEST0572
AUBES3688 AUSNP000052
AUSNP000077 AUSNP000038
AUBES2695
AUBES1792
AUEST0803
AUBES4321 AUEST0002
AUBES4724
AUNGS5096
AUBES2082
AUBES5344 AUBES3403
AUBES3404
AUBES1378
AUBES1597
AUBES5327
AUBES4310
AUBES4758
AUEST0900
AUBES4414
AUEST0220U AUEST0220L
AUEST0755
AUBES1075
AUEST0132
2
0.0
0.5
1.9
3.8
4.7
6.0
6.7
11.0
13.2
13.6
16.7
19.4
20.6
25.2
26.4
26.5
33.1
34.3
34.7
35.4
35.6
36.5
37.0
38.0
38.6
39.0
39.6
39.7
40.1
40.4
40.6
40.9
41.3
41.8
41.9
42.0
42.2
42.3
42.4
42.5
42.7
42.9
43.3
44.2
45.1
45.4
46.2
46.5
46.7
46.9
47.5
47.6
49.1
50.8
51.3
51.7
52.5
53.1
55.4
55.8
56.4
59.0
63.9
64.9
65.6
67.5
70.2
71.1
71.9
72.5
75.3
79.9
AUBES1263
AUBES1868
AUBES4268
AUBES4170
AUBES1947
AUBES3080
AUBES5080
AUBES4698
AUBES2884
AUBES2885
AUBES4165
AUBES1881
AUBES3952
AUEST0610
AUBES3395
AUBES2258
AUBES3626
AUBES3394
IpCG0007_U24
AUBES1586
AUBES5410
AUEST0771
AUBES3625
IpCG0134_U24
AUEST0714
AUBES4162
AUBES4789
AUBES4487
AUBES5146
AUBES1592
AUBES3627
AUBES5044
AUBES5300
AUBES3633
AUBES3628 IpCG0130_U24
AUBES4163 AUBES4434
AUBES2279 AUSNP000078
AUEST0514
AUBES4435 AUBES5387
AUBES3758
IpCG0182_U24 AUBES3082
AUBES3858 AUBES3036
AUBES3238
AUBES5001
AUBES1701
AUBES2676
IpCG0297_U24
AUBES3988 AUBES3978
AUBES4111
AUBES1842
AUBES2085 AUBES1853
AUBES1615
AUBES5241
AUBES5412
AUBES4269
AUEST0670
AUEST0836
AUBES4457
IpCG0293_U24
AUBES2036
AUBES4456
AUBES3183
AUBES3184
AUBES4633
AUBES3215
AUBES5046
AUBES3995
AUEST0106
AUEST0998
AUBES4858 AUBES5408
AUBES4859
AUBES2551
AUBES5332
AUEST0472
AUBES2152
3
0.0
0.1
4.6
6.5
9.0
10.8
25.2
30.2
30.9
32.0
33.4
34.4
37.1
38.6
42.2
42.8
44.9
47.4
48.3
48.5
49.4
51.8
54.0
54.1
54.3
57.7
58.4
58.6
59.4
61.0
61.7
62.1
62.4
62.9
63.0
63.6
63.7
64.6
64.8
65.0
65.2
65.4
65.5
65.6
66.1
66.2
66.5
67.5
68.0
68.8
71.2
72.3
72.8
73.2
73.3
73.9
74.3
74.4
74.5
74.6
74.8
74.9
75.2
75.4
75.9
76.2
76.9
77.0
77.6
79.8
79.9
80.8
81.4
81.6
81.7
82.0
83.1
83.8
85.0
99.9
AUEST0981
IpCG0136_U5
AUBES4824
AUBES1073
AUBES5154 AUBES5124
AUEST0815
AUEST0356B AUEST0356A
AUBES4476
AUBES4740
AUBES4718
AUBES4690
IpCG0124_U5
AUBES5331
AUBES3895
AUBES4395
AUBES5169
AUEST0783
IpCG0233_U5
AUBES1811
AUBES5048
AUBES5168
AUBES3536
AUBES1186
AUBES3065
AUEST0838
AUBES1590
AUBES3544
AUBES4343
AUBES3771
AUBES3074
AUBES2347
AUBES1846
AUBES2748
AUBES1742
AUBES1781
AUBES4784 AUBES4254
AUEST0829U AUBES4350
AUBES2595 AUSNP000072
AUEST0635U
AUBES5148 AUBES4950
AUBES2555 AUBES4702
AUBES4769
AUBES2052
AUBES2815
IpCG0290_U5 AUBES3646
AUBES5229 IpCG0088_U5
AUBES3879
AUBES4462 AUBES4271
AUBES1697 AUBES3327
AUBES4044
AUBES3139
AUBES3880 AUBES3647
AUBES3491
AUBES1626
AUBES2984
AUBES2097
AUBES5176
AUBES2550
AUBES4830
AUBES2926
AUBES2086 AUEST0753
AUBES5336
AUBES2423 AUBES4578
AUBES4593
AUBES5232 AUBES5259
AUBES4228
AUBES4876 AUBES5297
IgH_U5
AUBES4970
AUBES4875
AUBES3913
AUBES4502
AUBES2113
AUBES2091
AUBES2095
AUBES1963
AUBES4974 AUBES2411
AUBES5067 AUNGS5105
AUBES1381
AUBES4976
AUBES2958
AUBES3443
AUBES2837
AUBES3444 AUBES2421
AUBES4704
AUEST0406
AUBES2915
AUBES4620
4
0.0
17.1
18.1
23.2
26.4
28.6
31.5
35.4
35.5
37.1
37.7
38.4
45.3
50.0
50.8
52.3
52.4
52.8
53.5
54.1
54.5
55.0
55.5
55.7
55.8
56.0
56.2
56.4
56.8
57.0
57.3
57.5
57.6
57.8
57.9
58.0
58.1
58.2
58.3
58.4
58.5
58.6
58.8
58.9
59.0
59.3
59.5
59.6
59.7
59.8
60.2
60.3
60.8
61.1
61.5
61.7
61.8
62.2
63.0
65.3
66.4
69.4
74.0
77.0
86.1
87.9
88.3
AUBES1740
AUBES5273
AUBES2353
AUSNP000020 AUBES1795
AUBES4570
AUBES4936
AUBES1327
AUBES5203
AUBES5266
AUBES5234
AUBES4904
AUSNP000076
AUBES2399
IpCG0232_U6
IpCG0230_U6
IpCG0159_U12
IpCG0158_U6
AUBES5177
AUEST0678
AUBES4460
AUBES1153
IpCG0012_U6
AUBES4496
AUBES4257
AUEST0288
AUBES4565
AUEST0305
AUBES3780
AUEST0505 AUBES3638
AUBES4354
AUBES4743 AUBES1413
AUEST0508
AUBES2792 AUBES5397
AUBES3639
AUBES4036
AUBES2427
AUBES1841 AUBES1810
AUBES4084
AUBES1230 AUBES4083
AUBES4276 IpCG0192_U6
AUBES3012 AUBES4972
AUBES2459 AUBES2078
AUBES2403 AUBES2682
AUBES3576 AUBES2600
IpCG0309_U6 AUBES5296
AUBES5193 AUBES1103
AUBES4939 AUBES4681
AUBES4070 AUBES4071
AUBES3306 AUBES1926
AUBES4480
AUBES1555 AUBES3694
AUBES3171 AUBES4869
AUBES1617
AUBES3468 AUBES2084
AUBES4906
AUBES4881
AUBES2829
AUBES3376
AUBES4543
AUBES1319
AUBES5388 AUBES5134
AUBES4971 AUBES4708
AUBES2505 AUBES4270
AUBES4709
AUBES3760
AUBES3312
AUEST0424
AUBES2617
AUBES5409
AUBES3608
AUBES3607
AUBES5377
AUBES2921
AUBES3154
AUBES5267
AUBES5380
AUEST0052
AUEST0364
IpCG0273_U6
AUBES4498
AUBES2508
AUEST0960

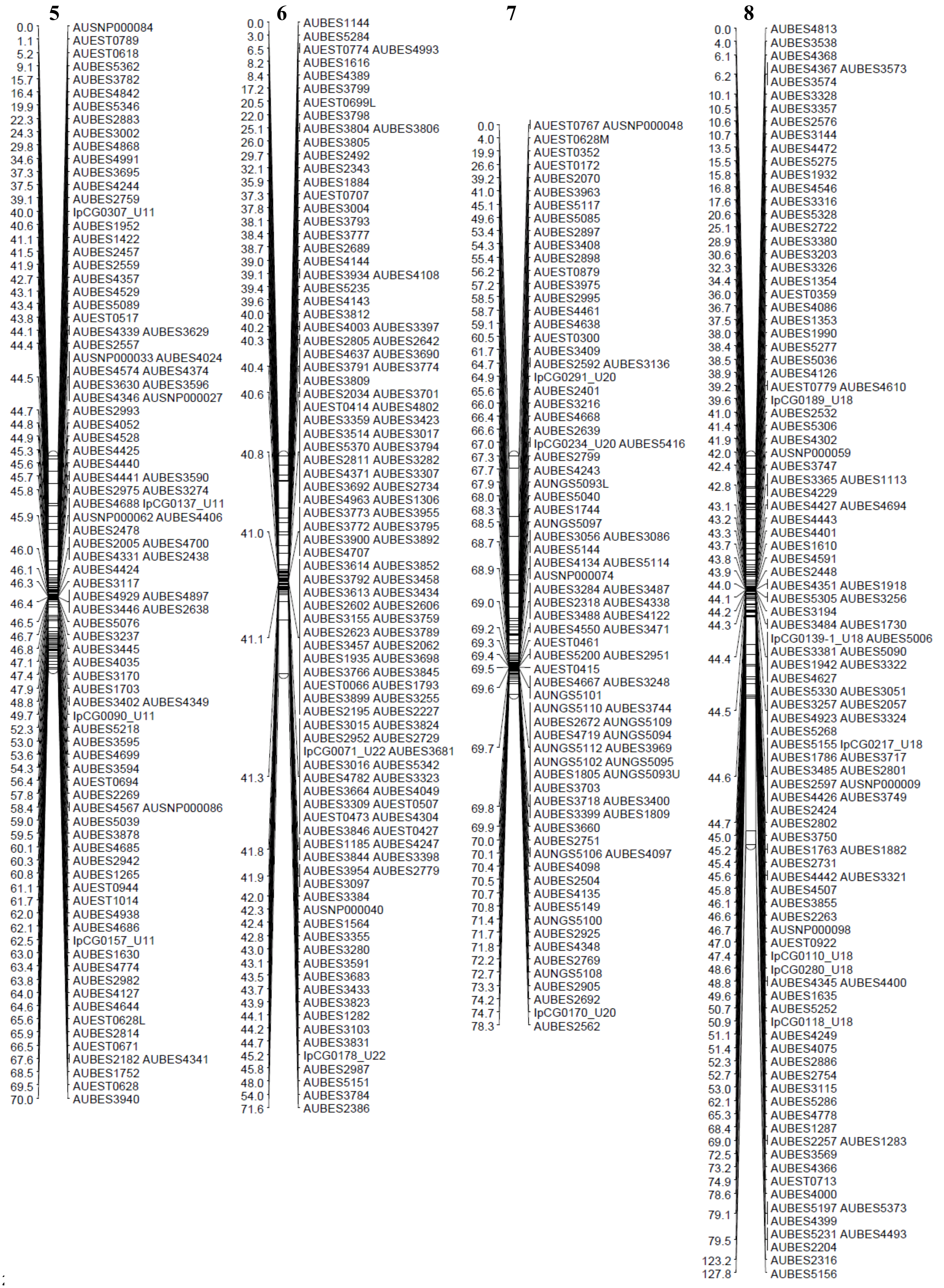
5
0.0
1.1
5.2
9.1
15.7
16.4
19.9
22.3
24.3
29.8
34.6
37.3
37.5
39.1
40.0
40.6
41.1
41.5
41.9
42.7
43.1
43.4
43.8
44.1
44.4
44.5
44.7
44.8
44.9
45.3
45.6
45.7
45.8
45.9
46.0
46.1
46.3
46.4
46.5
46.7
46.8
47.1
47.4
47.9
48.8
49.7
52.3
53.0
53.6
54.3
56.4
57.8
58.4
59.0
59.5
60.1
60.3
60.8
61.1
61.7
62.0
62.1
62.5
63.0
63.4
63.8
64.0
64.6
65.6
65.9
66.5
67.6
68.5
69.5
70.0
AUSNP000084
AUEST0789
AUEST0618
AUBES5362
AUBES3782
AUBES4842
AUBES5346
AUBES2883
AUBES3002
AUBES4868
AUBES4991
AUBES3695
AUBES4244
AUBES2759
IpCG0307_U11
AUBES1952
AUBES1422
AUBES2457
AUBES2559
AUBES4357
AUBES4529
AUBES5089
AUEST0517
AUBES4339 AUBES3629
AUBES2557
AUSNP000033 AUBES4024
AUBES4574 AUBES4374
AUBES3630 AUBES3596
AUBES4346 AUSNP000027
AUBES2993
AUBES4052
AUBES4528
AUBES4425
AUBES4440
AUBES4441 AUBES3590
AUBES2975 AUBES3274
AUBES4688 IpCG0137_U11
AUSNP000062 AUBES4406
AUBES2478
AUBES2005 AUBES4700
AUBES4331 AUBES2438
AUBES4424
AUBES3117
AUBES4929 AUBES4897
AUBES3446 AUBES2638
AUBES5076
AUBES3237
AUBES3445
AUBES4035
AUBES3170
AUBES1703
AUBES3402 AUBES4349
IpCG0090_U11
AUBES5218
AUBES3595
AUBES4699
AUBES3594
AUEST0694
AUBES2269
AUBES4567 AUSNP000086
AUBES5039
AUBES3878
AUBES4685
AUBES2942
AUBES1265
AUEST0944
AUEST1014
AUBES4938
AUBES4686
IpCG0157_U11
AUBES1630
AUBES4774
AUBES2982
AUBES4127
AUBES4644
AUEST0628L
AUBES2814
AUEST0671
AUBES2182 AUBES4341
AUBES1752
AUEST0628
AUBES3940
6
0.0
3.0
6.5
8.2
8.4
17.2
20.5
22.0
25.1
26.0
29.7
32.1
35.9
37.3
37.8
38.1
38.4
38.7
39.0
39.1
39.4
39.6
40.0
40.2
40.3
40.4
40.6
40.8
41.0
41.1
41.3
41.8
41.9
42.0
42.3
42.4
42.8
43.0
43.1
43.5
43.7
43.9
44.1
44.2
44.7
45.2
45.8
48.0
54.0
71.6
AUBES1144
AUBES5284
AUEST0774 AUBES4993
AUBES1616
AUBES4389
AUBES3799
AUEST0699L
AUBES3798
AUBES3804 AUBES3806
AUBES3805
AUBES2492
AUBES2343
AUBES1884
AUEST0707
AUBES3004
AUBES3793
AUBES3777
AUBES2689
AUBES4144
AUBES3934 AUBES4108
AUBES5235
AUBES4143
AUBES3812
AUBES4003 AUBES3397
AUBES2805 AUBES2642
AUBES4637 AUBES3690
AUBES3791 AUBES3774
AUBES3809
AUBES2034 AUBES3701
AUEST0414 AUBES4802
AUBES3359 AUBES3423
AUBES3514 AUBES3017
AUBES5370 AUBES3794
AUBES2811 AUBES3282
AUBES4371 AUBES3307
AUBES3692 AUBES2734
AUBES4963 AUBES1306
AUBES3773 AUBES3955
AUBES3772 AUBES3795
AUBES3900 AUBES3892
AUBES4707
AUBES3614 AUBES3852
AUBES3792 AUBES3458
AUBES3613 AUBES3434
AUBES2602 AUBES2606
AUBES3155 AUBES3759
AUBES2623 AUBES3789
AUBES3457 AUBES2062
AUBES1935 AUBES3698
AUBES3766 AUBES3845
AUEST0066 AUBES1793
AUBES3899 AUBES3255
AUBES2195 AUBES2227
AUBES3015 AUBES3824
AUBES2952 AUBES2729
IpCG0071_U22 AUBES3681
AUBES3016 AUBES5342
AUBES4782 AUBES3323
AUBES3664 AUBES4049
AUBES3309 AUEST0507
AUEST0473 AUBES4304
AUBES3846 AUEST0427
AUBES1185 AUBES4247
AUBES3844 AUBES3398
AUBES3954 AUBES2779
AUBES3097
AUBES3384
AUSNP000040
AUBES1564
AUBES3355
AUBES3280
AUBES3591
AUBES3683
AUBES3433
AUBES3823
AUBES1282
AUBES3103
AUBES3831
IpCG0178_U22
AUBES2987
AUBES5151
AUBES3784
AUBES2386
7
0.0
4.0
19.9
26.6
39.2
41.0
45.1
49.6
53.4
54.3
55.4
56.2
57.2
58.5
58.7
59.1
60.5
61.7
64.7
64.9
65.6
66.0
66.4
66.6
67.0
67.3
67.7
67.9
68.0
68.3
68.5
68.7
68.9
69.0
69.2
69.3
69.4
69.5
69.6
69.7
69.8
69.9
70.0
70.1
70.4
70.5
70.7
70.8
71.4
71.7
71.8
72.2
72.7
73.3
74.2
74.7
78.3
AUEST0767 AUSNP000048
AUEST0628M
AUEST0352
AUEST0172
AUBES2070
AUBES3963
AUBES5117
AUBES5085
AUBES2897
AUBES3408
AUBES2898
AUEST0879
AUBES3975
AUBES2995
AUBES4461
AUBES4638
AUEST0300
AUBES3409
AUBES2592 AUBES3136
IpCG0291_U20
AUBES2401
AUBES3216
AUBES4668
AUBES2639
IpCG0234_U20 AUBES5416
AUBES2799
AUBES4243
AUNGS5093L
AUBES5040
AUBES1744
AUNGS5097
AUBES3056 AUBES3086
AUBES5144
AUBES4134 AUBES5114
AUSNP000074
AUBES3284 AUBES3487
AUBES2318 AUBES4338
AUBES3488 AUBES4122
AUBES4550 AUBES3471
AUEST0461
AUBES5200 AUBES2951
AUEST0415
AUBES4667 AUBES3248
AUNGS5101
AUNGS5110 AUBES3744
AUBES2672 AUNGS5109
AUBES4719 AUNGS5094
AUNGS5112 AUBES3969
AUNGS5102 AUNGS5095
AUBES1805 AUNGS5093U
AUBES3703
AUBES3718 AUBES3400
AUBES3399 AUBES1809
AUBES3660
AUBES2751
AUNGS5106 AUBES4097
AUBES4098
AUBES2504
AUBES4135
AUBES5149
AUNGS5100
AUBES2925
AUBES4348
AUBES2769
AUNGS5108
AUBES2905
AUBES2692
IpCG0170_U20
AUBES2562
8
0.0
4.0
6.1
6.2
10.1
10.5
10.6
10.7
13.5
15.5
15.8
16.8
17.6
20.6
25.1
28.9
30.6
32.3
34.4
36.0
36.7
37.5
38.0
38.4
38.5
38.9
39.2
39.6
41.0
41.4
41.9
42.0
42.4
42.8
43.1
43.2
43.3
43.7
43.8
43.9
44.0
44.1
44.2
44.3
44.4
44.5
44.6
44.7
45.0
45.2
45.4
45.6
45.8
46.1
46.6
46.7
47.0
47.4
48.6
48.8
49.6
50.7
50.9
51.1
51.4
52.3
52.7
53.0
62.1
65.3
68.4
69.0
72.5
73.2
74.9
78.6
79.1
79.5
123.2
127.8
AUBES4813
AUBES3538
AUBES4368
AUBES4367 AUBES3573
AUBES3574
AUBES3328
AUBES3357
AUBES2576
AUBES3144
AUBES4472
AUBES5275
AUBES1932
AUBES4546
AUBES3316
AUBES5328
AUBES2722
AUBES3380
AUBES3203
AUBES3326
AUBES1354
AUEST0359
AUBES4086
AUBES1353
AUBES1990
AUBES5277
AUBES5036
AUBES4126
AUEST0779 AUBES4610
IpCG0189_U18
AUBES2532
AUBES5306
AUBES4302
AUSNP000059
AUBES3747
AUBES3365 AUBES1113
AUBES4229
AUBES4427 AUBES4694
AUBES4443
AUBES4401
AUBES1610
AUBES4591
AUBES2448
AUBES4351 AUBES1918
AUBES5305 AUBES3256
AUBES3194
AUBES3484 AUBES1730
IpCG0139-1_U18 AUBES5006
AUBES3381 AUBES5090
AUBES1942 AUBES3322
AUBES4627
AUBES5330 AUBES3051
AUBES3257 AUBES2057
AUBES4923 AUBES3324
AUBES5268
AUBES5155 IpCG0217_U18
AUBES1786 AUBES3717
AUBES3485 AUBES2801
AUBES2597 AUSNP000009
AUBES4426 AUBES3749
AUBES2424
AUBES2802
AUBES3750
AUBES1763 AUBES1882
AUBES2731
AUBES4442 AUBES3321
AUBES4507
AUBES3855
AUBES2263
AUSNP000098
AUEST0922
IpCG0110_U18
IpCG0280_U18
AUBES4345 AUBES4400
AUBES1635
AUBES5252
IpCG0118_U18
AUBES4249
AUBES4075
AUBES2886
AUBES2754
AUBES3115
AUBES5286
AUBES4778
AUBES1287
AUBES2257 AUBES1283
AUBES3569
AUBES4366
AUEST0713
AUBES4000
AUBES5197 AUBES5373
AUBES4399
AUBES5231 AUBES4493
AUBES2204
AUBES2316
AUBES5156

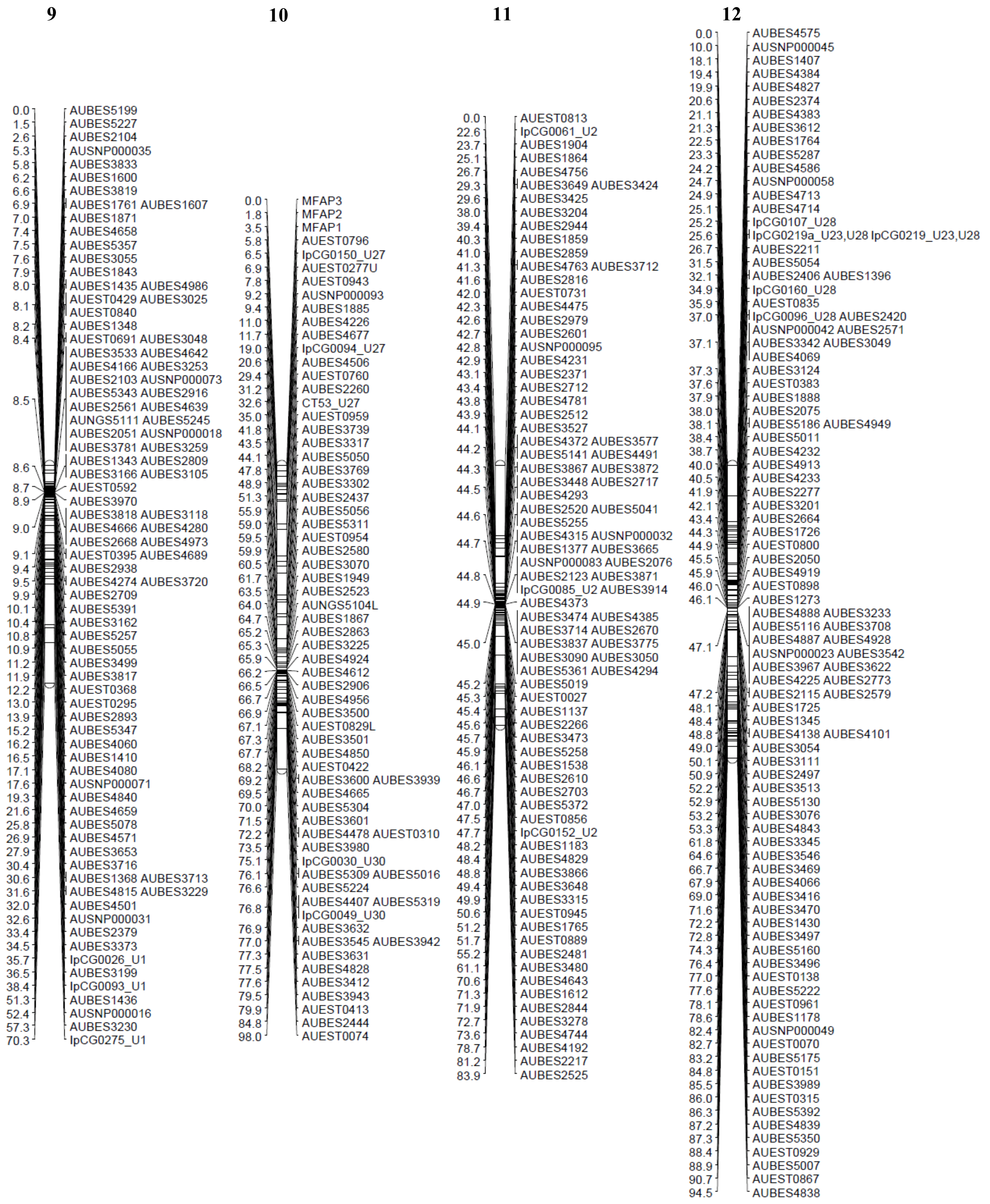
9
0.0
1.5
2.6
5.3
5.8
6.2
6.6
6.9
7.0
7.4
7.5
7.6
7.9
8.0
8.1
8.2
8.4
8.5
8.6
8.7
8.9
9.0
9.1
9.4
9.5
9.9
10.1
10.4
10.8
10.9
11.2
11.9
12.2
13.0
13.9
15.2
16.2
16.5
17.1
17.6
19.3
21.6
25.8
26.9
27.9
30.4
30.6
31.6
32.0
32.6
33.4
34.5
35.7
36.5
38.4
51.3
52.4
57.3
70.3
AUBES5199
AUBES5227
AUBES2104
AUSNP000035
AUBES3833
AUBES1600
AUBES3819
AUBES1761 AUBES1607
AUBES1871
AUBES4658
AUBES5357
AUBES3055
AUBES1843
AUBES1435 AUBES4986
AUEST0429 AUBES3025
AUEST0840
AUBES1348
AUEST0691 AUBES3048
AUBES3533 AUBES4642
AUBES4166 AUBES3253
AUBES2103 AUSNP000073
AUBES5343 AUBES2916
AUBES2561 AUBES4639
AUNGS5111 AUBES5245
AUBES2051 AUSNP000018
AUBES3781 AUBES3259
AUBES1343 AUBES2809
AUBES3166 AUBES3105
AUEST0592
AUBES3970
AUBES3818 AUBES3118
AUBES4666 AUBES4280
AUBES2668 AUBES4973
AUEST0395 AUBES4689
AUBES2938
AUBES4274 AUBES3720
AUBES2709
AUBES5391
AUBES3162
AUBES5257
AUBES5055
AUBES3499
AUBES3817
AUEST0368
AUEST0295
AUBES2893
AUBES5347
AUBES4060
AUBES1410
AUBES4080
AUSNP000071
AUBES4840
AUBES4659
AUBES5078
AUBES4571
AUBES3653
AUBES3716
AUBES1368 AUBES3713
AUBES4815 AUBES3229
AUBES4501
AUSNP000031
AUBES2379
AUBES3373
IpCG0026_U1
AUBES3199
IpCG0093_U1
AUBES1436
AUSNP000016
AUBES3230
IpCG0275_U1
10
0.0
1.8
3.5
5.8
6.5
6.9
7.8
9.2
9.4
11.0
11.7
19.0
20.6
29.4
31.2
32.6
35.0
41.8
43.5
44.1
47.8
48.9
51.3
55.9
59.0
59.5
59.9
60.5
61.7
63.5
64.0
64.7
65.2
65.3
65.9
66.2
66.5
66.7
66.9
67.1
67.3
67.7
68.2
69.2
69.5
70.0
71.5
72.2
73.5
75.1
76.1
76.6
76.8
76.9
77.0
77.3
77.5
77.6
79.5
79.9
84.8
98.0
MFAP3
MFAP2
MFAP1
AUEST0796
IpCG0150_U27
AUEST0277U
AUEST0943
AUSNP000093
AUBES1885
AUBES4226
AUBES4677
IpCG0094_U27
AUBES4506
AUEST0760
AUBES2260
CT53_U27
AUEST0959
AUBES3739
AUBES3317
AUBES5050
AUBES3769
AUBES3302
AUBES2437
AUBES5056
AUBES5311
AUEST0954
AUBES2580
AUBES3070
AUBES1949
AUBES2523
AUNGS5104L
AUBES1867
AUBES2863
AUBES3225
AUBES4924
AUBES4612
AUBES2906
AUBES4956
AUBES3500
AUEST0829L
AUBES3501
AUBES4850
AUEST0422
AUBES3600 AUBES3939
AUBES4665
AUBES5304
AUBES3601
AUBES4478 AUEST0310
AUBES3980
IpCG0030_U30
AUBES5309 AUBES5016
AUBES5224
AUBES4407 AUBES5319
IpCG0049_U30
AUBES3632
AUBES3545 AUBES3942
AUBES3631
AUBES4828
AUBES3412
AUBES3943
AUEST0413
AUBES2444
AUEST0074
11
0.0
22.6
23.7
25.1
26.7
29.3
29.6
38.0
39.4
40.3
41.0
41.3
41.6
42.0
42.3
42.6
42.7
42.8
42.9
43.1
43.4
43.8
43.9
44.1
44.2
44.3
44.5
44.6
44.7
44.8
44.9
45.0
45.2
45.3
45.4
45.6
45.7
45.9
46.1
46.6
46.7
47.0
47.5
47.7
48.2
48.4
48.8
49.4
49.9
50.6
51.2
51.7
55.2
61.1
70.6
71.3
71.9
72.7
73.6
78.7
81.2
83.9
AUEST0813
IpCG0061_U2
AUBES1904
AUBES1864
AUBES4756
AUBES3649 AUBES3424
AUBES3425
AUBES3204
AUBES2944
AUBES1859
AUBES2859
AUBES4763 AUBES3712
AUBES2816
AUEST0731
AUBES4475
AUBES2979
AUBES2601
AUSNP000095
AUBES4231
AUBES2371
AUBES2712
AUBES4781
AUBES2512
AUBES3527
AUBES4372 AUBES3577
AUBES5141 AUBES4491
AUBES3867 AUBES3872
AUBES3448 AUBES2717
AUBES4293
AUBES2520 AUBES5041
AUBES5255
AUBES4315 AUSNP000032
AUBES1377 AUBES3665
AUSNP000083 AUBES2076
AUBES2123 AUBES3871
IpCG0085_U2 AUBES3914
AUBES4373
AUBES3474 AUBES4385
AUBES3714 AUBES2670
AUBES3837 AUBES3775
AUBES3090 AUBES3050
AUBES5361 AUBES4294
AUBES5019
AUEST0027
AUBES1137
AUBES2266
AUBES3473
AUBES5258
AUBES1538
AUBES2610
AUBES2703
AUBES5372
AUEST0856
IpCG0152_U2
AUBES1183
AUBES4829
AUBES3866
AUBES3648
AUBES3315
AUEST0945
AUBES1765
AUEST0889
AUBES2481
AUBES3480
AUBES4643
AUBES1612
AUBES2844
AUBES3278
AUBES4744
AUBES4192
AUBES2217
AUBES2525
12
0.0
10.0
18.1
19.4
19.9
20.6
21.1
21.3
22.5
23.3
24.2
24.7
24.9
25.1
25.2
25.6
26.7
31.5
32.1
34.9
35.9
37.0
37.1
37.3
37.6
37.9
38.0
38.1
38.4
38.7
40.0
40.5
41.9
42.1
43.4
44.3
44.9
45.5
45.9
46.0
46.1
47.1
47.2
48.1
48.4
48.8
49.0
50.1
50.9
52.2
52.9
53.2
53.3
61.8
64.6
66.7
67.9
69.0
71.6
72.2
72.8
74.3
76.4
77.0
77.6
78.1
78.6
82.4
82.7
83.2
84.8
85.5
86.0
86.3
87.2
87.3
88.4
88.9
90.7
94.5
AUBES4575
AUSNP000045
AUBES1407
AUBES4384
AUBES4827
AUBES2374
AUBES4383
AUBES3612
AUBES1764
AUBES5287
AUBES4586
AUSNP000058
AUBES4713
AUBES4714
IpCG0107_U28
IpCG0219a_U23,U28 IpCG0219_U23,U28
AUBES2211
AUBES5054
AUBES2406 AUBES1396
IpCG0160_U28
AUEST0835
IpCG0096_U28 AUBES2420
AUSNP000042 AUBES2571
AUBES3342 AUBES3049
AUBES4069
AUBES3124
AUEST0383
AUBES1888
AUBES2075
AUBES5186 AUBES4949
AUBES5011
AUBES4232
AUBES4913
AUBES4233
AUBES2277
AUBES3201
AUBES2664
AUBES1726
AUEST0800
AUBES2050
AUBES4919
AUEST0898
AUBES1273
AUBES4888 AUBES3233
AUBES5116 AUBES3708
AUBES4887 AUBES4928
AUSNP000023 AUBES3542
AUBES3967 AUBES3622
AUBES4225 AUBES2773
AUBES2115 AUBES2579
AUBES1725
AUBES1345
AUBES4138 AUBES4101
AUBES3054
AUBES3111
AUBES2497
AUBES3513
AUBES5130
AUBES3076
AUBES4843
AUBES3345
AUBES3546
AUBES3469
AUBES4066
AUBES3416
AUBES3470
AUBES1430
AUBES3497
AUBES5160
AUBES3496
AUEST0138
AUBES5222
AUEST0961
AUBES1178
AUSNP000049
AUEST0070
AUBES5175
AUEST0151
AUBES3989
AUEST0315
AUBES5392
AUBES4839
AUBES5350
AUEST0929
AUBES5007
AUEST0867
AUBES4838

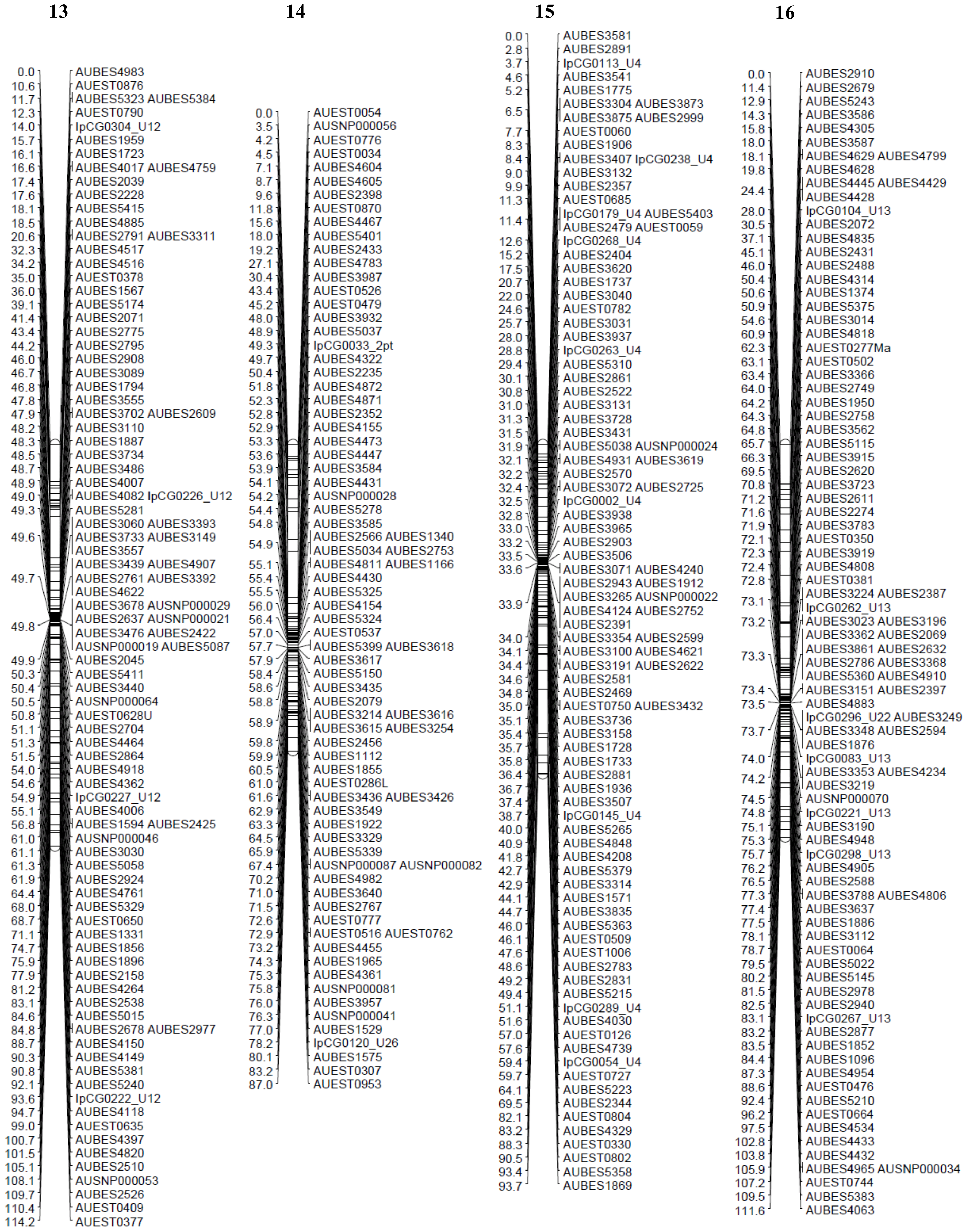
13
0.0
10.6
11.7
12.3
14.0
15.7
16.1
16.6
17.4
17.6
18.1
18.5
20.6
32.3
34.2
35.0
36.0
39.1
41.4
43.4
44.2
46.0
46.7
46.8
47.8
47.9
48.2
48.3
48.5
48.7
48.9
49.0
49.3
49.6
49.7
49.8
49.9
50.3
50.4
50.5
50.8
51.1
51.3
51.5
54.0
54.6
54.9
55.1
56.8
61.0
61.1
61.3
61.9
64.4
68.0
68.7
71.1
74.7
75.9
77.9
81.2
83.1
84.6
84.8
88.7
90.3
90.8
92.1
93.6
94.7
99.0
100.7
101.5
105.1
108.1
109.7
110.4
114.2
AUBES4983
AUEST0876
AUBES5323 AUBES5384
AUEST0790
IpCG0304_U12
AUBES1959
AUBES1723
AUBES4017 AUBES4759
AUBES2039
AUBES2228
AUBES5415
AUBES4885
AUBES2791 AUBES3311
AUBES4517
AUBES4516
AUEST0378
AUBES1567
AUBES5174
AUBES2071
AUBES2775
AUBES2795
AUBES2908
AUBES3089
AUBES1794
AUBES3555
AUBES3702 AUBES2609
AUBES3110
AUBES1887
AUBES3734
AUBES3486
AUBES4007
AUBES4082 IpCG0226_U12
AUBES5281
AUBES3060 AUBES3393
AUBES3733 AUBES3149
AUBES3557
AUBES3439 AUBES4907
AUBES2761 AUBES3392
AUBES4622
AUBES3678 AUSNP000029
AUBES2637 AUSNP000021
AUBES3476 AUBES2422
AUSNP000019 AUBES5087
AUBES2045
AUBES5411
AUBES3440
AUSNP000064
AUEST0628U
AUBES2704
AUBES4464
AUBES2864
AUBES4918
AUBES4362
IpCG0227_U12
AUBES4006
AUBES1594 AUBES2425
AUSNP000046
AUBES3030
AUBES5058
AUBES2924
AUBES4761
AUBES5329
AUEST0650
AUBES1331
AUBES1856
AUBES1896
AUBES2158
AUBES4264
AUBES2538
AUBES5015
AUBES2678 AUBES2977
AUBES4150
AUBES4149
AUBES5381
AUBES5240
IpCG0222_U12
AUBES4118
AUEST0635
AUBES4397
AUBES4820
AUBES2510
AUSNP000053
AUBES2526
AUEST0409
AUEST0377
14
0.0
3.5
4.2
4.5
7.1
8.7
9.6
11.8
15.6
18.0
19.2
27.1
30.4
43.4
45.2
48.0
48.9
49.3
49.7
50.4
51.8
52.3
52.8
52.9
53.3
53.6
53.9
54.1
54.2
54.4
54.8
54.9
55.1
55.4
55.5
56.0
56.4
57.0
57.7
57.9
58.4
58.6
58.8
58.9
59.8
59.9
60.5
61.0
61.6
62.9
63.3
64.5
65.9
67.4
70.2
71.0
71.5
72.6
72.9
73.2
74.3
75.3
75.8
76.0
76.3
77.0
78.2
80.1
83.2
87.0
AUEST0054
AUSNP000056
AUEST0776
AUEST0034
AUBES4604
AUBES4605
AUBES2398
AUEST0870
AUBES4467
AUBES5401
AUBES2433
AUBES4783
AUBES3987
AUEST0526
AUEST0479
AUBES3932
AUBES5037
IpCG0033_2pt
AUBES4322
AUBES2235
AUBES4872
AUBES4871
AUBES2352
AUBES4155
AUBES4473
AUBES4447
AUBES3584
AUBES4431
AUSNP000028
AUBES5278
AUBES3585
AUBES2566 AUBES1340
AUBES5034 AUBES2753
AUBES4811 AUBES1166
AUBES4430
AUBES5325
AUBES4154
AUBES5324
AUEST0537
AUBES5399 AUBES3618
AUBES3617
AUBES5150
AUBES3435
AUBES2079
AUBES3214 AUBES3616
AUBES3615 AUBES3254
AUBES2456
AUBES1112
AUBES1855
AUEST0286L
AUBES3436 AUBES3426
AUBES3549
AUBES1922
AUBES3329
AUBES5339
AUSNP000087 AUSNP000082
AUBES4982
AUBES3640
AUBES2767
AUEST0777
AUEST0516 AUEST0762
AUBES4455
AUBES1965
AUBES4361
AUSNP000081
AUBES3957
AUSNP000041
AUBES1529
IpCG0120_U26
AUBES1575
AUEST0307
AUEST0953
15
0.0
2.8
3.7
4.6
5.2
6.5
7.7
8.3
8.4
9.0
9.9
11.3
11.4
12.6
15.2
17.5
20.7
22.0
24.6
25.7
28.0
28.8
29.4
30.1
30.8
31.0
31.3
31.5
31.9
32.1
32.2
32.4
32.5
32.8
33.0
33.2
33.5
33.6
33.9
34.0
34.1
34.4
34.6
34.8
35.0
35.1
35.4
35.7
35.8
36.4
36.7
37.4
38.7
40.0
40.9
41.8
42.7
42.9
44.1
44.7
46.0
46.1
47.6
48.6
49.2
49.4
51.1
51.6
57.0
57.6
59.4
59.7
64.1
69.5
82.1
83.2
88.3
90.5
93.4
93.7
AUBES3581
AUBES2891
IpCG0113_U4
AUBES3541
AUBES1775
AUBES3304 AUBES3873
AUBES3875 AUBES2999
AUEST0060
AUBES1906
AUBES3407 IpCG0238_U4
AUBES3132
AUBES2357
AUEST0685
IpCG0179_U4 AUBES5403
AUBES2479 AUEST0059
IpCG0268_U4
AUBES2404
AUBES3620
AUBES1737
AUBES3040
AUEST0782
AUBES3031
AUBES3937
IpCG0263_U4
AUBES5310
AUBES2861
AUBES2522
AUBES3131
AUBES3728
AUBES3431
AUBES5038 AUSNP000024
AUBES4931 AUBES3619
AUBES2570
AUBES3072 AUBES2725
IpCG0002_U4
AUBES3938
AUBES3965
AUBES2903
AUBES3506
AUBES3071 AUBES4240
AUBES2943 AUBES1912
AUBES3265 AUSNP000022
AUBES4124 AUBES2752
AUBES2391
AUBES3354 AUBES2599
AUBES3100 AUBES4621
AUBES3191 AUBES2622
AUBES2581
AUBES2469
AUEST0750 AUBES3432
AUBES3736
AUBES3158
AUBES1728
AUBES1733
AUBES2881
AUBES1936
AUBES3507
IpCG0145_U4
AUBES5265
AUBES4848
AUBES4208
AUBES5379
AUBES3314
AUBES1571
AUBES3835
AUBES5363
AUEST0509
AUEST1006
AUBES2783
AUBES2831
AUBES5215
IpCG0289_U4
AUBES4030
AUEST0126
AUBES4739
IpCG0054_U4
AUEST0727
AUBES5223
AUBES2344
AUEST0804
AUBES4329
AUEST0330
AUEST0802
AUBES5358
AUBES1869
16
0.0
11.4
12.9
14.3
15.8
18.0
18.1
19.8
24.4
28.0
30.5
37.1
45.1
46.0
50.4
50.6
50.9
54.6
60.9
62.3
63.1
63.4
64.0
64.2
64.3
64.8
65.7
66.3
69.5
70.8
71.2
71.6
71.9
72.1
72.3
72.4
72.8
73.1
73.2
73.3
73.4
73.5
73.7
74.0
74.2
74.5
74.8
75.1
75.3
75.7
76.2
76.5
77.3
77.4
77.5
78.1
78.7
79.5
80.2
81.5
82.5
83.1
83.2
83.5
84.4
87.3
88.6
92.4
96.2
97.5
102.8
103.8
105.9
107.2
109.5
111.6
AUBES2910
AUBES2679
AUBES5243
AUBES3586
AUBES4305
AUBES3587
AUBES4629 AUBES4799
AUBES4628
AUBES4445 AUBES4429
AUBES4428
IpCG0104_U13
AUBES2072
AUBES4835
AUBES2431
AUBES2488
AUBES4314
AUBES1374
AUBES5375
AUBES3014
AUBES4818
AUEST0277Ma
AUEST0502
AUBES3366
AUBES2749
AUBES1950
AUBES2758
AUBES3562
AUBES5115
AUBES3915
AUBES2620
AUBES3723
AUBES2611
AUBES2274
AUBES3783
AUEST0350
AUBES3919
AUBES4808
AUEST0381
AUBES3224 AUBES2387
IpCG0262_U13
AUBES3023 AUBES3196
AUBES3362 AUBES2069
AUBES3861 AUBES2632
AUBES2786 AUBES3368
AUBES5360 AUBES4910
AUBES3151 AUBES2397
AUBES4883
IpCG0296_U22 AUBES3249
AUBES3348 AUBES2594
AUBES1876
IpCG0083_U13
AUBES3353 AUBES4234
AUBES3219
AUSNP000070
IpCG0221_U13
AUBES3190
AUBES4948
IpCG0298_U13
AUBES4905
AUBES2588
AUBES3788 AUBES4806
AUBES3637
AUBES1886
AUBES3112
AUEST0064
AUBES5022
AUBES5145
AUBES2978
AUBES2940
IpCG0267_U13
AUBES2877
AUBES1852
AUBES1096
AUBES4954
AUEST0476
AUBES5210
AUEST0664
AUBES4534
AUBES4433
AUBES4432
AUBES4965 AUSNP000034
AUEST0744
AUBES5383
AUBES4063

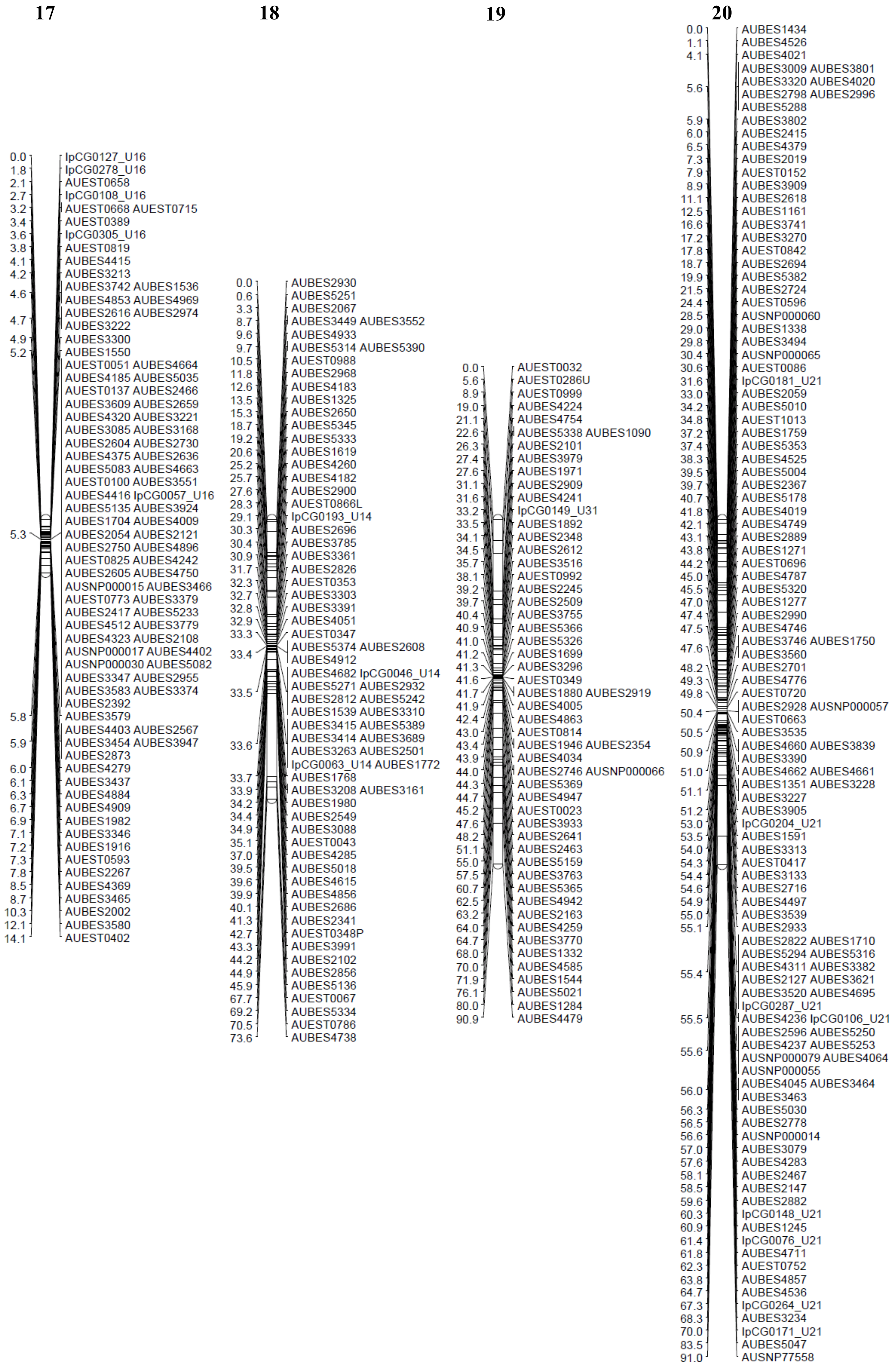
17
0.0
1.8
2.1
2.7
3.2
3.4
3.6
3.8
4.1
4.2
4.6
4.7
4.9
5.2
5.3
5.8
5.9
6.0
6.1
6.3
6.7
6.9
7.1
7.2
7.3
7.8
8.5
8.7
10.3
12.1
14.1
IpCG0127_U16
IpCG0278_U16
AUEST0658
IpCG0108_U16
AUEST0668 AUEST0715
AUEST0389
IpCG0305_U16
AUEST0819
AUBES4415
AUBES3213
AUBES3742 AUBES1536
AUBES4853 AUBES4969
AUBES2616 AUBES2974
AUBES3222
AUBES3300
AUBES1550
AUEST0051 AUBES4664
AUBES4185 AUBES5035
AUEST0137 AUBES2466
AUBES3609 AUBES2659
AUBES4320 AUBES3221
AUBES3085 AUBES3168
AUBES2604 AUBES2730
AUBES4375 AUBES2636
AUBES5083 AUBES4663
AUEST0100 AUBES3551
AUBES4416 IpCG0057_U16
AUBES5135 AUBES3924
AUBES1704 AUBES4009
AUBES2054 AUBES2121
AUBES2750 AUBES4896
AUEST0825 AUBES4242
AUBES2605 AUBES4750
AUSNP000015 AUBES3466
AUEST0773 AUBES3379
AUBES2417 AUBES5233
AUBES4512 AUBES3779
AUBES4323 AUBES2108
AUSNP000017 AUBES4402
AUSNP000030 AUBES5082
AUBES3347 AUBES2955
AUBES3583 AUBES3374
AUBES2392
AUBES3579
AUBES4403 AUBES2567
AUBES3454 AUBES3947
AUBES2873
AUBES4279
AUBES3437
AUBES4884
AUBES4909
AUBES1982
AUBES3346
AUBES1916
AUEST0593
AUBES2267
AUBES4369
AUBES3465
AUBES2002
AUBES3580
AUEST0402
18
0.0
0.6
3.3
8.7
9.6
9.7
10.5
11.8
12.6
13.5
15.3
18.7
19.2
20.6
25.2
25.7
27.6
28.3
29.1
30.3
30.4
30.9
31.7
32.3
32.7
32.8
32.9
33.3
33.4
33.5
33.6
33.7
33.9
34.2
34.4
34.9
35.1
37.0
39.5
39.6
39.9
40.1
41.3
42.7
43.3
44.2
44.9
45.9
67.7
69.2
70.5
73.6
AUBES2930
AUBES5251
AUBES2067
AUBES3449 AUBES3552
AUBES4933
AUBES5314 AUBES5390
AUEST0988
AUBES2968
AUBES4183
AUBES1325
AUBES2650
AUBES5345
AUBES5333
AUBES1619
AUBES4260
AUBES4182
AUBES2900
AUEST0866L
IpCG0193_U14
AUBES2696
AUBES3785
AUBES3361
AUBES2826
AUEST0353
AUBES3303
AUBES3391
AUBES4051
AUEST0347
AUBES5374 AUBES2608
AUBES4912
AUBES4682 IpCG0046_U14
AUBES5271 AUBES2932
AUBES2812 AUBES5242
AUBES1539 AUBES3310
AUBES3415 AUBES5389
AUBES3414 AUBES3689
AUBES3263 AUBES2501
IpCG0063_U14 AUBES1772
AUBES1768
AUBES3208 AUBES3161
AUBES1980
AUBES2549
AUBES3088
AUEST0043
AUBES4285
AUBES5018
AUBES4615
AUBES4856
AUBES2686
AUBES2341
AUEST0348P
AUBES3991
AUBES2102
AUBES2856
AUBES5136
AUEST0067
AUBES5334
AUEST0786
AUBES4738
19
0.0
5.6
8.9
19.0
21.1
22.6
26.3
27.4
27.6
31.1
31.6
33.2
33.5
34.1
34.5
35.7
38.1
39.2
39.7
40.4
40.9
41.0
41.2
41.3
41.6
41.7
41.9
42.4
43.0
43.4
43.9
44.0
44.3
44.7
45.2
47.6
48.2
51.1
55.0
57.5
60.7
62.5
63.2
64.0
64.7
68.0
70.0
71.9
76.1
80.0
90.9
AUEST0032
AUEST0286U
AUEST0999
AUBES4224
AUBES4754
AUBES5338 AUBES1090
AUBES2101
AUBES3979
AUBES1971
AUBES2909
AUBES4241
IpCG0149_U31
AUBES1892
AUBES2348
AUBES2612
AUBES3516
AUEST0992
AUBES2245
AUBES2509
AUBES3755
AUBES5366
AUBES5326
AUBES1699
AUBES3296
AUEST0349
AUBES1880 AUBES2919
AUBES4005
AUBES4863
AUEST0814
AUBES1946 AUBES2354
AUBES4034
AUBES2746 AUSNP000066
AUBES5369
AUBES4947
AUEST0023
AUBES3933
AUBES2641
AUBES2463
AUBES5159
AUBES3763
AUBES5365
AUBES4942
AUBES2163
AUBES4259
AUBES3770
AUBES1332
AUBES4585
AUBES1544
AUBES5021
AUBES1284
AUBES4479
20
0.0
1.1
4.1
5.6
5.9
6.0
6.5
7.3
7.9
8.9
11.1
12.5
16.6
17.2
17.8
18.7
19.9
21.5
24.4
28.5
29.0
29.8
30.4
30.6
31.6
33.0
34.2
34.8
37.2
37.4
38.3
39.5
39.7
40.7
41.8
42.1
43.1
43.8
44.2
45.0
45.5
47.0
47.4
47.5
47.6
48.2
49.3
49.8
50.4
50.5
50.9
51.0
51.1
51.2
53.0
53.5
54.0
54.3
54.4
54.6
54.9
55.0
55.1
55.4
55.5
55.6
56.0
56.3
56.5
56.6
57.0
57.6
58.1
58.5
59.6
60.3
60.9
61.4
61.8
62.3
63.8
64.7
67.3
68.3
70.0
83.5
91.0
AUBES1434
AUBES4526
AUBES4021
AUBES3009 AUBES3801
AUBES3320 AUBES4020
AUBES2798 AUBES2996
AUBES5288
AUBES3802
AUBES2415
AUBES4379
AUBES2019
AUEST0152
AUBES3909
AUBES2618
AUBES1161
AUBES3741
AUBES3270
AUEST0842
AUBES2694
AUBES5382
AUBES2724
AUEST0596
AUSNP000060
AUBES1338
AUBES3494
AUSNP000065
AUEST0086
IpCG0181_U21
AUBES2059
AUBES5010
AUEST1013
AUBES1759
AUBES5353
AUBES4525
AUBES5004
AUBES2367
AUBES5178
AUBES4019
AUBES4749
AUBES2889
AUBES1271
AUEST0696
AUBES4787
AUBES5320
AUBES1277
AUBES2990
AUBES4746
AUBES3746 AUBES1750
AUBES3560
AUBES2701
AUBES4776
AUEST0720
AUBES2928 AUSNP000057
AUEST0663
AUBES3535
AUBES4660 AUBES3839
AUBES3390
AUBES4662 AUBES4661
AUBES1351 AUBES3228
AUBES3227
AUBES3905
IpCG0204_U21
AUBES1591
AUBES3313
AUEST0417
AUBES3133
AUBES2716
AUBES4497
AUBES3539
AUBES2933
AUBES2822 AUBES1710
AUBES5294 AUBES5316
AUBES4311 AUBES3382
AUBES2127 AUBES3621
AUBES3520 AUBES4695
IpCG0287_U21
AUBES4236 IpCG0106_U21
AUBES2596 AUBES5250
AUBES4237 AUBES5253
AUSNP000079 AUBES4064
AUSNP000055
AUBES4045 AUBES3464
AUBES3463
AUBES5030
AUBES2778
AUSNP000014
AUBES3079
AUBES4283
AUBES2467
AUBES2147
AUBES2882
IpCG0148_U21
AUBES1245
IpCG0076_U21
AUBES4711
AUEST0752
AUBES4857
AUBES4536
IpCG0264_U21
AUBES3234
IpCG0171_U21
AUBES5047
AUSNP77558

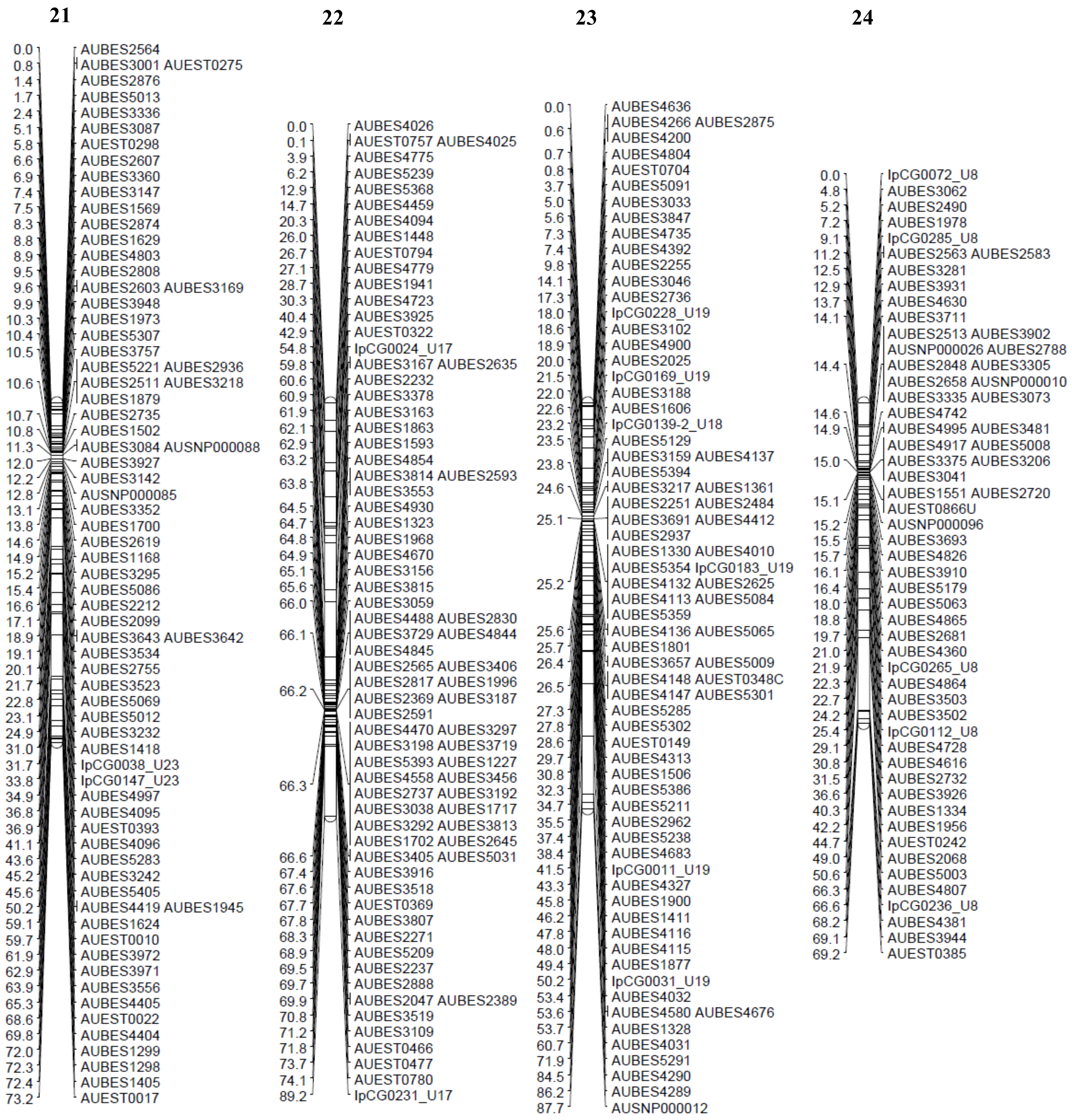
21
0.0
0.8
1.4
1.7
2.4
5.1
5.8
6.6
6.9
7.4
7.5
8.3
8.8
8.9
9.5
9.6
9.9
10.3
10.4
10.5
10.6
10.7
10.8
11.3
12.0
12.2
12.8
13.1
13.8
14.6
14.9
15.2
15.4
16.6
17.1
18.9
19.1
20.1
21.7
22.8
23.1
24.9
31.0
31.7
33.8
34.9
36.8
36.9
41.1
43.6
45.2
45.6
50.2
59.1
59.7
61.9
62.9
63.9
65.3
68.6
69.8
72.0
72.3
72.4
73.2
AUBES2564
AUBES3001 AUEST0275
AUBES2876
AUBES5013
AUBES3336
AUBES3087
AUEST0298
AUBES2607
AUBES3360
AUBES3147
AUBES1569
AUBES2874
AUBES1629
AUBES4803
AUBES2808
AUBES2603 AUBES3169
AUBES3948
AUBES1973
AUBES5307
AUBES3757
AUBES5221 AUBES2936
AUBES2511 AUBES3218
AUBES1879
AUBES2735
AUBES1502
AUBES3084 AUSNP000088
AUBES3927
AUBES3142
AUSNP000085
AUBES3352
AUBES1700
AUBES2619
AUBES1168
AUBES3295
AUBES5086
AUBES2212
AUBES2099
AUBES3643 AUBES3642
AUBES3534
AUBES2755
AUBES3523
AUBES5069
AUBES5012
AUBES3232
AUBES1418
IpCG0038_U23
IpCG0147_U23
AUBES4997
AUBES4095
AUEST0393
AUBES4096
AUBES5283
AUBES3242
AUBES5405
AUBES4419 AUBES1945
AUBES1624
AUEST0010
AUBES3972
AUBES3971
AUBES3556
AUBES4405
AUEST0022
AUBES4404
AUBES1299
AUBES1298
AUBES1405
AUEST0017
22
0.0
0.1
3.9
6.2
12.9
14.7
20.3
26.0
26.7
27.1
28.7
30.3
40.4
42.9
54.8
59.8
60.6
60.9
61.9
62.1
62.9
63.2
63.8
64.5
64.7
64.8
64.9
65.1
65.6
66.0
66.1
66.2
66.3
66.6
67.4
67.6
67.7
67.8
68.3
68.9
69.5
69.7
69.9
70.8
71.2
71.8
73.7
74.1
89.2
AUBES4026
AUEST0757 AUBES4025
AUBES4775
AUBES5239
AUBES5368
AUBES4459
AUBES4094
AUBES1448
AUEST0794
AUBES4779
AUBES1941
AUBES4723
AUBES3925
AUEST0322
IpCG0024_U17
AUBES3167 AUBES2635
AUBES2232
AUBES3378
AUBES3163
AUBES1863
AUBES1593
AUBES4854
AUBES3814 AUBES2593
AUBES3553
AUBES4930
AUBES1323
AUBES1968
AUBES4670
AUBES3156
AUBES3815
AUBES3059
AUBES4488 AUBES2830
AUBES3729 AUBES4844
AUBES4845
AUBES2565 AUBES3406
AUBES2817 AUBES1996
AUBES2369 AUBES3187
AUBES2591
AUBES4470 AUBES3297
AUBES3198 AUBES3719
AUBES5393 AUBES1227
AUBES4558 AUBES3456
AUBES2737 AUBES3192
AUBES3038 AUBES1717
AUBES3292 AUBES3813
AUBES1702 AUBES2645
AUBES3405 AUBES5031
AUBES3916
AUBES3518
AUEST0369
AUBES3807
AUBES2271
AUBES5209
AUBES2237
AUBES2888
AUBES2047 AUBES2389
AUBES3519
AUBES3109
AUEST0466
AUEST0477
AUEST0780
IpCG0231_U17
23
0.0
0.6
0.7
0.8
3.7
5.0
5.6
7.3
7.4
9.8
14.1
17.3
18.0
18.6
18.9
20.0
21.5
22.0
22.6
23.2
23.5
23.8
24.6
25.1
25.2
25.6
25.7
26.4
26.5
27.3
27.8
28.6
29.7
30.8
32.3
34.7
35.5
37.4
38.4
41.5
43.3
45.8
46.2
47.8
48.0
49.4
50.2
53.4
53.6
53.7
60.7
71.9
84.5
86.2
87.7
AUBES4636
AUBES4266 AUBES2875
AUBES4200
AUBES4804
AUEST0704
AUBES5091
AUBES3033
AUBES3847
AUBES4735
AUBES4392
AUBES2255
AUBES3046
AUBES2736
IpCG0228_U19
AUBES3102
AUBES4900
AUBES2025
IpCG0169_U19
AUBES3188
AUBES1606
IpCG0139-2_U18
AUBES5129
AUBES3159 AUBES4137
AUBES5394
AUBES3217 AUBES1361
AUBES2251 AUBES2484
AUBES3691 AUBES4412
AUBES2937
AUBES1330 AUBES4010
AUBES5354 IpCG0183_U19
AUBES4132 AUBES2625
AUBES4113 AUBES5084
AUBES5359
AUBES4136 AUBES5065
AUBES1801
AUBES3657 AUBES5009
AUBES4148 AUEST0348C
AUBES4147 AUBES5301
AUBES5285
AUBES5302
AUEST0149
AUBES4313
AUBES1506
AUBES5386
AUBES5211
AUBES2962
AUBES5238
AUBES4683
IpCG0011_U19
AUBES4327
AUBES1900
AUBES1411
AUBES4116
AUBES4115
AUBES1877
IpCG0031_U19
AUBES4032
AUBES4580 AUBES4676
AUBES1328
AUBES4031
AUBES5291
AUBES4290
AUBES4289
AUSNP000012
24
0.0
4.8
5.2
7.2
9.1
11.2
12.5
12.9
13.7
14.1
14.4
14.6
14.9
15.0
15.1
15.2
15.5
15.7
16.1
16.4
18.0
18.8
19.7
21.0
21.9
22.3
22.7
24.2
25.4
29.1
30.8
31.5
36.6
40.3
42.2
44.7
49.0
50.6
66.3
66.6
68.2
69.1
69.2
IpCG0072_U8
AUBES3062
AUBES2490
AUBES1978
IpCG0285_U8
AUBES2563 AUBES2583
AUBES3281
AUBES3931
AUBES4630
AUBES3711
AUBES2513 AUBES3902
AUSNP000026 AUBES2788
AUBES2848 AUBES3305
AUBES2658 AUSNP000010
AUBES3335 AUBES3073
AUBES4742
AUBES4995 AUBES3481
AUBES4917 AUBES5008
AUBES3375 AUBES3206
AUBES3041
AUBES1551 AUBES2720
AUEST0866U
AUSNP000096
AUBES3693
AUBES4826
AUBES3910
AUBES5179
AUBES5063
AUBES4865
AUBES2681
AUBES4360
IpCG0265_U8
AUBES4864
AUBES3503
AUBES3502
IpCG0112_U8
AUBES4728
AUBES4616
AUBES2732
AUBES3926
AUBES1334
AUBES1956
AUEST0242
AUBES2068
AUBES5003
AUBES4807
IpCG0236_U8
AUBES4381
AUBES3944
AUEST0385

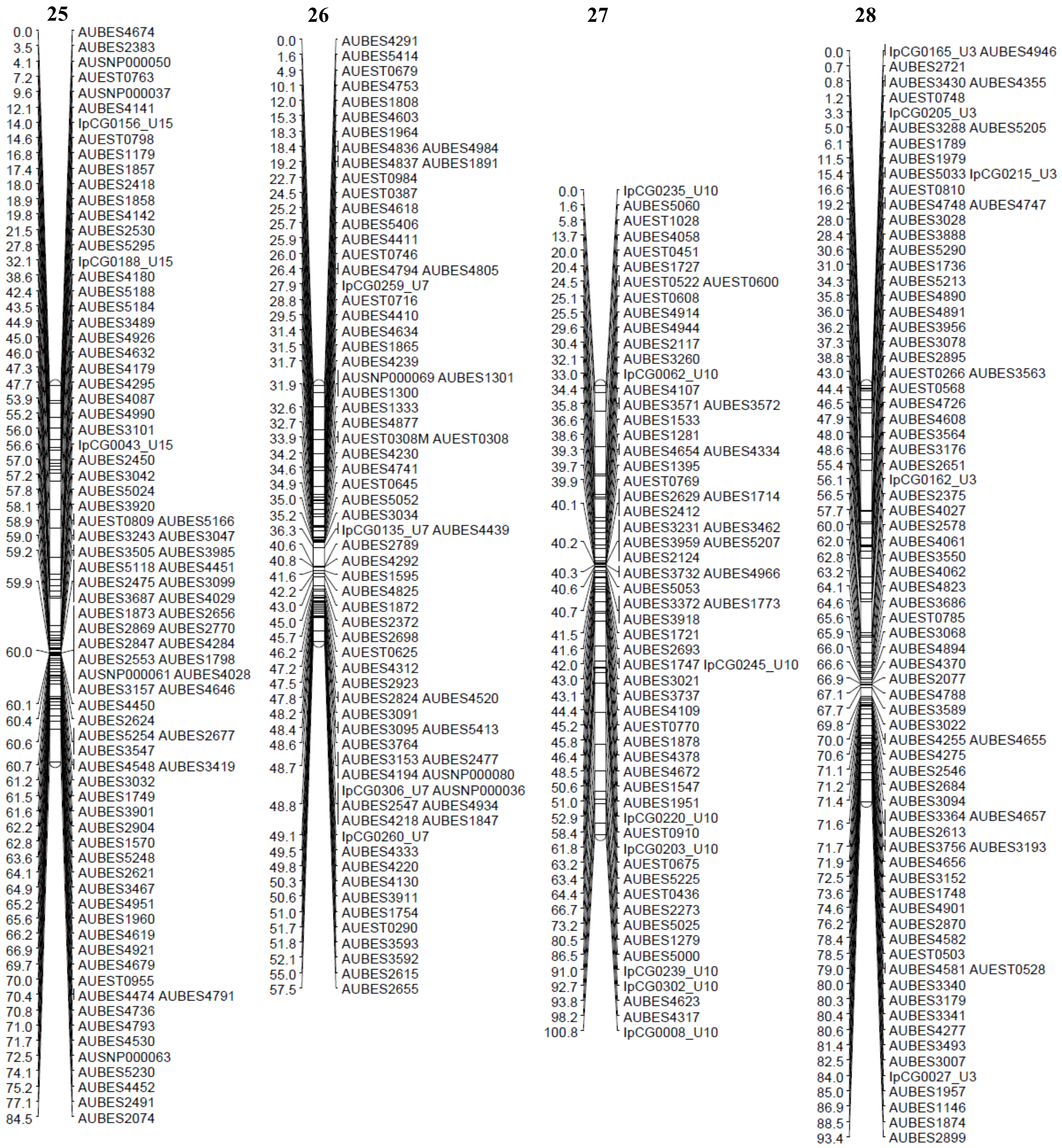
25
0.0 3.5 4.1 7.2 9.6 12.1 14.0 14.6 16.8 17.4 18.0 18.9 19.8 21.5 27.8 32.1 38.6 42.4 43.5 44.9 45.0 46.0 47.3 47.7 53.9 55.2 56.0 56.6 57.0 57.2 57.8 58.1 58.9 59.0 59.2 59.9 60.0 60.1 60.4 60.6 60.7 61.2 61.5 61.6 62.2 62.8 63.6 64.1 64.9 65.2 65.6 66.2 66.9 69.7 70.0 70.4 70.8 71.0 71.7 72.5 74.1 75.2 77.1 84.5
AUBES4674
AUBES2383
AUSNP000050
AUEST0763
AUSNP000037
AUBES4141
IpCG0156_U15
AUEST0798
AUBES1179
AUBES1857
AUBES2418
AUBES1858
AUBES4142
AUBES2530
AUBES5295
IpCG0188_U15
AUBES4180
AUBES5188
AUBES5184
AUBES3489
AUBES4926
AUBES4632
AUBES4179
AUBES4295
AUBES4087
AUBES4990
AUBES3101
IpCG0043_U15
AUBES2450
AUBES3042
AUBES5024
AUBES3920
AUEST0809 AUBES5166
AUBES3243 AUBES3047
AUBES3505 AUBES3985
AUBES5118 AUBES4451
AUBES2475 AUBES3099
AUBES3687 AUBES4029
AUBES1873 AUBES2656
AUBES2869 AUBES2770
AUBES2847 AUBES4284
AUBES2553 AUBES1798
AUSNP000061 AUBES4028
AUBES3157 AUBES4646
AUBES4450
AUBES2624
AUBES5254 AUBES2677
AUBES3547
AUBES4548 AUBES3419
AUBES3032
AUBES1749
AUBES3901
AUBES2904
AUBES1570
AUBES5248
AUBES2621
AUBES3467
AUBES4951
AUBES1960
AUBES4619
AUBES4921
AUBES4679
AUEST0955
AUBES4474 AUBES4791
AUBES4736
AUBES4793
AUBES4530
AUSNP000063
AUBES5230
AUBES4452
AUBES2491
AUBES2074
26
0.0 1.6 4.9 10.1 12.0 15.3 18.3 18.4 19.2 22.7 24.5 25.2 25.7 25.9 26.0 26.4 27.9 28.8 29.5 31.4 31.5 31.7 31.9 32.6 32.7 33.9 34.2 34.6 34.9 35.0 35.2 36.3 40.6 40.8 41.6 42.2 43.0 45.0 45.7 46.2 47.2 47.5 47.8 48.2 48.4 48.6 48.7 48.8 49.1 49.5 49.8 50.3 50.6 51.0 51.7 51.8 52.1 55.0 57.5
AUBES4291
AUBES5414
AUEST0679
AUBES4753
AUBES1808
AUBES4603
AUBES1964
AUBES4836 AUBES4984
AUBES4837 AUBES1891
AUEST0984
AUEST0387
AUBES4618
AUBES5406
AUBES4411
AUEST0746
AUBES4794 AUBES4805
IpCG0259_U7
AUEST0716
AUBES4410
AUBES4634
AUBES1865
AUBES4239
AUSNP000069 AUBES1301
AUBES1300
AUBES1333
AUBES4877
AUEST0308M AUEST0308
AUBES4230
AUBES4741
AUEST0645
AUBES5052
AUBES3034
IpCG0135_U7 AUBES4439
AUBES2789
AUBES4292
AUBES1595
AUBES4825
AUBES1872
AUBES2372
AUBES2698
AUEST0625
AUBES4312
AUBES2923
AUBES2824 AUBES4520
AUBES3091
AUBES3095 AUBES5413
AUBES3764
AUBES3153 AUBES2477
AUBES4194 AUSNP000080
IpCG0306_U7 AUSNP000036
AUBES2547 AUBES4934
AUBES4218 AUBES1847
IpCG0260_U7
AUBES4333
AUBES4220
AUBES4130
AUBES3911
AUBES1754
AUEST0290
AUBES3593
AUBES3592
AUBES2615
AUBES2655
27
0.0 1.6 5.8 13.7 20.0 20.4 24.5 25.1 25.5 29.6 30.4 32.1 33.0 34.4 35.8 36.6 38.6 39.3 39.7 39.9 40.1 40.2 40.3 40.6 40.7 41.5 41.6 42.0 43.0 43.1 44.4 45.2 45.8 46.4 48.5 50.6 51.0 52.9 58.4 61.8 63.2 63.4 64.4 66.7 73.2 80.5 86.5 91.0 92.7 93.8 98.2 100.8
IpCG0235_U10
AUBES5060
AUEST1028
AUBES4058
AUEST0451
AUBES1727
AUEST0522 AUEST0600
AUEST0608
AUBES4914
AUBES4944
AUBES2117
AUBES3260
IpCG0062_U10
AUBES4107
AUBES3571 AUBES3572
AUBES1533
AUBES1281
AUBES4654 AUBES4334
AUBES1395
AUEST0769
AUBES2629 AUBES1714
AUBES2412
AUBES3231 AUBES3462
AUBES3959 AUBES5207
AUBES2124
AUBES3732 AUBES4966
AUBES5053
AUBES3372 AUBES1773
AUBES3918
AUBES1721
AUBES2693
AUBES1747 IpCG0245_U10
AUBES3021
AUBES3737
AUBES4109
AUEST0770
AUBES1878
AUBES4378
AUBES4672
AUBES1547
AUBES1951
IpCG0220_U10
AUEST0910
IpCG0203_U10
AUEST0675
AUBES5225
AUEST0436
AUBES2273
AUBES5025
AUBES1279
AUBES5000
IpCG0239_U10
IpCG0302_U10
AUBES4623
AUBES4317
IpCG0008_U10
28
0.0 0.7 0.8 1.2 3.3 5.0 6.1 11.5 15.4 16.6 19.2 28.0 28.4 30.6 31.0 34.3 35.8 36.0 36.2 37.3 38.8 43.0 44.4 46.5 47.9 48.0 48.6 55.4 56.1 56.5 57.7 60.0 62.0 62.8 63.2 64.1 64.6 65.6 65.9 66.0 66.6 66.9 67.1 67.7 69.8 70.0 70.6 71.1 71.2 71.4 71.6 71.7 71.9 72.5 73.6 74.6 76.2 78.4 78.5 79.0 80.0 80.3 80.4 80.6 81.4 82.5 84.0 85.0 86.9 88.5 93.4
IpCG0165_U3 AUBES4946
AUBES2721
AUBES3430 AUBES4355
AUEST0748
IpCG0205_U3
AUBES3288 AUBES5205
AUBES1789
AUBES1979
AUBES5033 IpCG0215_U3
AUEST0810
AUBES4748 AUBES4747
AUBES3028
AUBES3888
AUBES5290
AUBES1736
AUBES5213
AUBES4890
AUBES4891
AUBES3956
AUBES3078
AUBES2895
AUEST0266 AUBES3563
AUEST0568
AUBES4726
AUBES4608
AUBES3564
AUBES3176
AUBES2651
IpCG0162_U3
AUBES2375
AUBES4027
AUBES2578
AUBES4061
AUBES3550
AUBES4062
AUBES4823
AUBES3686
AUEST0785
AUBES3068
AUBES4894
AUBES4370
AUBES2077
AUBES4788
AUBES3589
AUBES3022
AUBES4255 AUBES4655
AUBES4275
AUBES2546
AUBES2684
AUBES3094
AUBES3364 AUBES4657
AUBES2613
AUBES3756 AUBES3193
AUBES4656
AUBES3152
AUBES1748
AUBES4901
AUBES2870
AUBES4582
AUEST0503
AUBES4581 AUEST0528
AUBES3340
AUBES3179
AUBES3341
AUBES4277
AUBES3493
AUBES3007
IpCG0027_U3
AUBES1957
AUBES1146
AUBES1874
AUBES2899

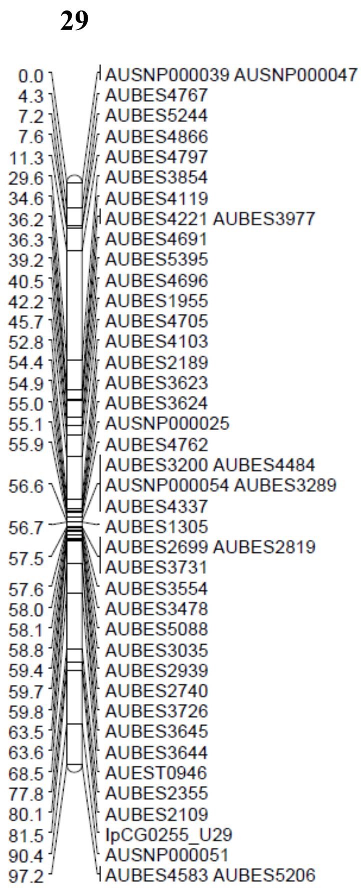

Figure S1 A sex-averaged linkage map of channel catfish. Genetic map distance was given in centimorgans (Kosambi's mapping function) to the left of the markers positions. The vertical straight lines indicated markers placed at the same positions.
